# Clinicopathological Characteristics and Prediction of Overall Survival and Death Within 2 Years in Diffuse Large B-Cell Lymphoma Based on Histological Images and Deep Learning

**DOI:** 10.3390/biomedicines14051134

**Published:** 2026-05-17

**Authors:** Joaquim Carreras

**Affiliations:** Department of Pathology, School of Medicine, Tokai University, 143 Shimokasuya, Isehara 259-1193, Kanagawa, Japan; joaquim.carreras@tokai.ac.jp

**Keywords:** DLBCL, prognosis, overall survival, deep learning, CNN, XAI, computer vision, AI, reactive lymphoid tissue

## Abstract

**Background**: Diffuse large B-cell lymphoma (DLBCL) is one of the most frequent lymphomas. To date, it is not possible to identify which DLBCL patients will have an aggressive clinical evolution only by using hematoxylin and eosin (H&E) histological images. **Methods**: This study predicted the prognosis of DLBCL using H&E images, computer vision and deep learning. The series included 114 DLBCL cases, split into 2 prognostic groups according to overall survival, and 44 cases of reactive lymphoid tissue. **Results**: The curve fitting and slope analysis showed a point of inflection at 2 years (24 months), which differentiated patients with aggressive clinical evolution (“Dead < 2 years”, b1 = −0.024) from the rest with moderate clinical evolution (“Others”, b1 = −0.003). Twenty different convolutional neural networks (CNNs) were used, and explainable artificial intelligence (XAI) was also applied. The final model based on DarkNet-19 predicted prognosis groups with high performance (test set accuracy = 96.3%). The other performance parameters were precision (94.5%), recall (95.0%), false positive rate (3.1%), specificity (96.9%), and F1 score (94.7%). XAI, including grad-CAM, occlusion sensitivity, and image-LIME, confirmed that the CNN focused on the correct areas. Hybrid partitioning to prevent information leakage with patient-based analysis, image classification between DLBCL and 44 cases of reactive lymphoid tissue, and hyperparameter tuning were also successfully performed. Correlation with the clinicopathological characteristics found that the Dead < 2 years group was correlated with stage III–IV, International Prognostic Index (IPI) High + High/intermediate, progressive disease, non-GCB cell-of-origin, CD10−, BCL2+, and Epstein–Barr virus (EBER)+. Analysis of the microenvironment, immune checkpoint, cell cycle, and germinal center markers showed that Dead < 2 years had higher IL10, PD-L1, and CD163 levels and lower E2F1 protein expression. No differences were found for Ki67, CSF1R, CASP8, TNFAIP8, LMO2, MYC, MDM2, CDK6, and TP53 markers at a quantitative level. **Conclusions**: The DLBCL overall survival can be predicted using H&E histological images and deep learning using the 2-year (24 months) point (similar to POD24). This trained CNN can be used as a pretrained model for transfer learning in the future.

## 1. Introduction

Diffuse large B-cell lymphoma (DLBCL) is one of the most frequent histological subtypes of non-Hodgkin lymphoma (NHL), accounting for approximately 25% of adult NHL cases [1]. Most patients present with a rapidly enlarging symptomatic mass with nodal enlargement. In approximately 60% of the cases, DLBCL presents with advanced-stage (III or IV) disease and high serum lactate dehydrogenase (LDH) levels, while 30% present with fever, weight loss, night sweats, and bone marrow infiltration [2,3,4,5,6,7]. DLBCL is cured in 60% of the cases with current therapy, particularly in patients who achieve a complete response with first-line treatment. However, in 40% of the cases, the clinical evolution is unfavorable [5,8,9].

Deep learning has been applied to DLBCL research in recent years. The types of research include computer vision of radiological images from PET/CT [10,11,12,13] and other studies based on histological images, gene expression and omics approaches [14,15,16,17,18,19,20,21,22]. Chava Perry et al. developed an image-based deep learning method for detecting high-grade B-cell lymphomas directly from hematoxylin and eosin images [19]. Vrabac Damir et al. used several morphological features to compute deep learning from an annotated digital DLBCL image set that included H&E and immunohistochemical stains of CD10, BCL6, MUM1, BCL2 and MYC [22]. Zheng Li et al. developed and validated a deep learning model based on clinical and histological features to predict the outcome of DLBCL [15]. Naji Hussein et al. developed the HoLy-Net, a method for the segmentation of histological images of DLBCL based on HoVer-Net deep learning [23]. Dongguang Li et al. established a highly accurate deep learning platform, consisting of multiple convolutional neural networks, to classify pathologic images using smaller datasets of DLBCL and other non-DLBCL images [24]. Therefore, in the field of DLBCL research, deep learning is a hot topic.

DLBCL is a heterogeneous clinicopathological entity. It is derived from germinal center B cells (centroblasts) or post-germinal activated B cells (immunoblasts) [25,26]. The tumor cells of DLBCL are large (e.g., nuclei twice the size of a small lymphocyte and larger than the nucleus of a macrophage) [25,26,27,28,29,30,31,32]. Centroblasts are large, noncleaved cells with round or oval nuclei, vesicular chromatin, often with multiple peripheral nucleoli, and a narrow rim of basophilic cytoplasm [25,26,27,28,29,30,31,32]. Immunoblasts are usually larger cells with prominent nucleoli and more abundant cytoplasm, often with plasmacytoid characteristics. Some cases are mixed, and other morphological variants exist [25,26,28,29]. The pathogenesis of DLBCL is complex and shows a heterogeneous landscape [1]. Our study will use deep learning to analyze the H&E images of DLBCL, focusing on the characteristics of neoplastic B lymphocytes in an unbiased manner and the traditional DLBCL classification, including centroblastic, immunoblastic, anaplastic, etc.

DLBCL not otherwise specified (NOS) is defined as having a mature B-cell phenotype and large cell morphology, but having none of the criteria that define specific large B-cell lymphoma subtypes. Using either immunohistochemistry or gene expression studies of DLBCL, the cell-of-origin (COO) status can be determined, including germinal center B-cell (GCB) versus activated B-cell (ABC) subtypes [2,5]. Other large B-cell lymphoma subtypes include primary mediastinal, T-cell/histiocyte-rich, plasmablastic, primary cutaneous leg-type, immune-privileged sites, intravascular, associated with chronic inflammation, IRF4 rearranged, ALK-positive, with 11q aberration, primary effusion lymphoma, and Epstein-Barr virus (EBV)-positive DLBCL [2,5].

Nodal DLBCL can spread to other organs, such as the liver, kidneys, lungs, bone marrow, and central nervous system. Extranodal/extramedullary involvement is frequently present in early-stage disease (stage I/II). The most frequent primary extranodal presentation is in the gastrointestinal tract, but virtually any tissue could be affected, including the testis, bone, thyroid, salivary glands, tonsils, and skin [1,33,34,35].

Several prognostic models are used in DLBCL. The International Prognostic Index (IPI) and its variants are the main prognostic variables routinely used. The original IPI included the following factors: age > 60 years, high serum LDH, Eastern Cooperative Oncology Group (ECOG) performance status ≥ 2, clinical stage III–IV, and number of extranodal sites > 1, all associated with poor prognosis [36]. The COO status, determined by immunohistochemistry using CD10, BCL6, and MUM1 or by gene expression profiling, correlated with non-GCB/ABC-like subtypes, which have a poorer prognosis for patients [36,37,38,39,40,41,42]. *MYC* rearrangement is seen in 5–15% of the cases and can be associated with *BCL2* and *BCL6* rearrangements [43]. Double-hit *MYC* and *BCL2* cases have a worse prognosis [2,5,44,45,46,47,48,49,50]. Other techniques, such as deep sequencing, have confirmed the DLBCL heterogeneity and identified driver mutations with different clinical outcomes [2,5,6,32,51,52,53].

The histological characteristics of the tissue reflect the proteomic, transcriptomic, and genomic pathological background. Several studies have focused on each of these aspects, but elucidating the prognosis of DLBCL based only on hematoxylin and eosin (H&E) staining as “raw” histological images remains unresolved. This study aimed to predict the prognosis of patients with DLBCL based only on the histological evaluation of H&E staining. The 2-year timepoint, which is similar to the progression of disease within 24 months (POD24), shows a change in the slope of the survival curve. To date, a comprehensive analysis of DLBCL using the 2-year cutoff timepoint has not been fully performed. Therefore, the DLBCL cases were classified according to their overall survival: “aggressive” and “moderate”. Using several models, the prognosis was predicted with high performance, and several explainable artificial intelligence (XAI) techniques were used to visually highlight the regions that are most important for the deep neural network’s classification decision.

## 2. Materials and Methods

### 2.1. Patients and Samples

A series of 114 patients with a histological diagnosis of conventional diffuse large B-cell lymphoma (DLBCL), consecutive cases from 2007 to 2011, were selected from the Department of Pathology, Tokai University, School of Medicine. The cases were diagnosed according to the current classifications of hematolymphoid neoplasia [2,5,25], which included the evaluation of hematoxylin and eosin (H&E), immunophenotype, and molecular techniques when required [2,5,6]. No specific exclusion criteria were applied, but samples of minimum size (above 5 mm) were necessary to construct a tissue microarray. All cases were de novo DLBCL, and all were diagnostic biopsies.

Supplementary Materials shows examples of images.

This study was conducted following the guidelines of the Declaration of Helsinki of the World Medical Association and ethical principles for medical research involving human participants. The Institutional Review Board of Tokai University approved the study (IRB20-156).

Immune microenvironment data were retrieved from our previous publications [54,55,56], and immunohistochemistry was reanalyzed. For CD163, IL10, and PD-L1, new immunohistochemistry (IHC) was performed as the number of cases in this study increased from previous publications. The IHC methodology, including primary antibody details, is shown in Appendix B. Notably, the immunohistochemical data was not included as input in the deep learning analysis but was used to describe different clinicopathological characteristics of the series.

### 2.2. Overall Survival Curve Analysis

**Figure 1:**
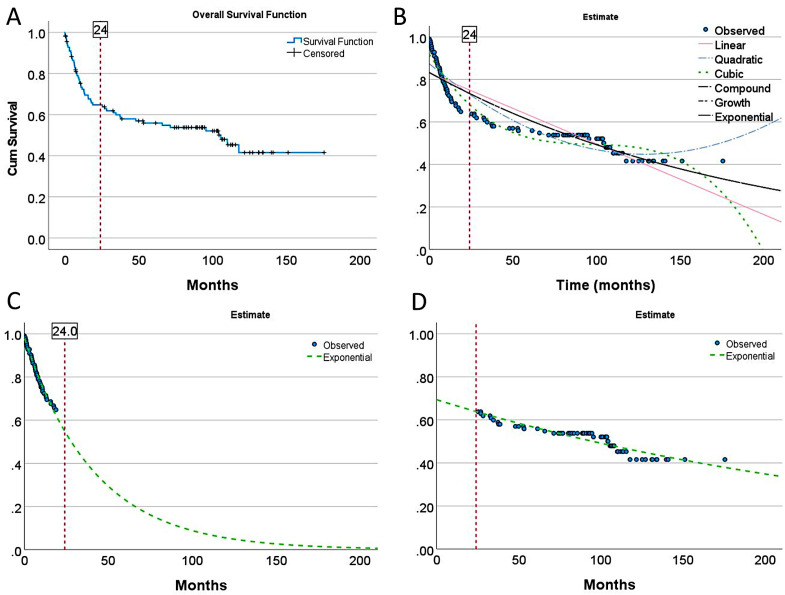
Overall survival groups. (**A**) The cases were grouped according to their survival based on the slope of the Kaplan–Meier survival curve and the point of inflection at the 2-year mark: “aggressive” (mainly patients who died within the first 24 months (Dead < 2 years); b1 = −0.024) and more “moderate” clinical evolution (b1 = −0.003). (**B**) A detailed analysis was performed using curve estimation. (**C**,**D**) An exponential curve estimation allowed for quantifying the different slopes of each group.

Analysis of the overall survival plot identified 2 regions with different prognoses based on the 2-year point. The rationale is that at the 2-year mark, the slope of the overall survival curve changes (point of inflection), changing from aggressive (b1 = −0.024; y = 0.98 * exp(−0.024 * x); exponential equation, R^2^ = 0.985, *p* < 0.001) to moderate clinical behavior (b1 = −0.003; y = 0.69 * exp(−0.003 * x); exponential equation, R^2^ = 0.868, *p* < 0.001) (Table 1). The “aggressive” group was mainly characterized by a death event before 2 years (*p* < 0.001). A logistic regression was performed to ascertain the effect of group variables on the likelihood that participants died. The aggressive group was 15.4 times more likely to exhibit a death event than the moderate group (*p* < 0.001) (Table 1 and Table 2) (Figure 1).

### 2.3. Survival Groups

Based on the overall survival curve and the 2-year inflection point, 2 survival groups were defined: the aggressive clinical behavior group, characterized by a death event before 2 years (“Dead < 2 years”), and the “Others” group. The “Others” group included all cases with a follow-up of >2 years, both censored (alive) or with a death event. The “Others” group initially also included 8 cases of alive patients that had a follow-up of less than 2 years. Notably, in a subsequent analysis, these 8 cases were excluded from the study.

### 2.4. Deep Learning Image Classification

Whole-tissue sections were stained with H&E and digitized using a slide scanner (NanoZoomer S360, C13220-01, Hamamatsu Photonics K.K., Hamamatsu, Japan). The whole neoplastic areas were identified, digitally extracted at 20× magnification and 150 dpi, and split into image patches of 224 × 224 × 3 resolution. After splitting, image patches of sizes different from 224 × 224 and those with less than 80% of tissue were discarded. As previously described, non-diagnostic image patches were also discarded [57,58,59,60]. The image patches are anonymized. After splitting the images, the patches were filtered. The criteria were as follows: (1) image patches of only 224 × 224 size; (2) image patches of more than 5–31 KB that contain at least 20–30% of viable tissue; (3) image patches with diagnostic areas; (4) image patches without artifacts, including broken tissue, folded areas, incorrectly stained tissue, and smashed/crushed tissue. Steps 3 and 4 were manually curated by a pathology specialist (MD, PhD).

**Figure A7:**
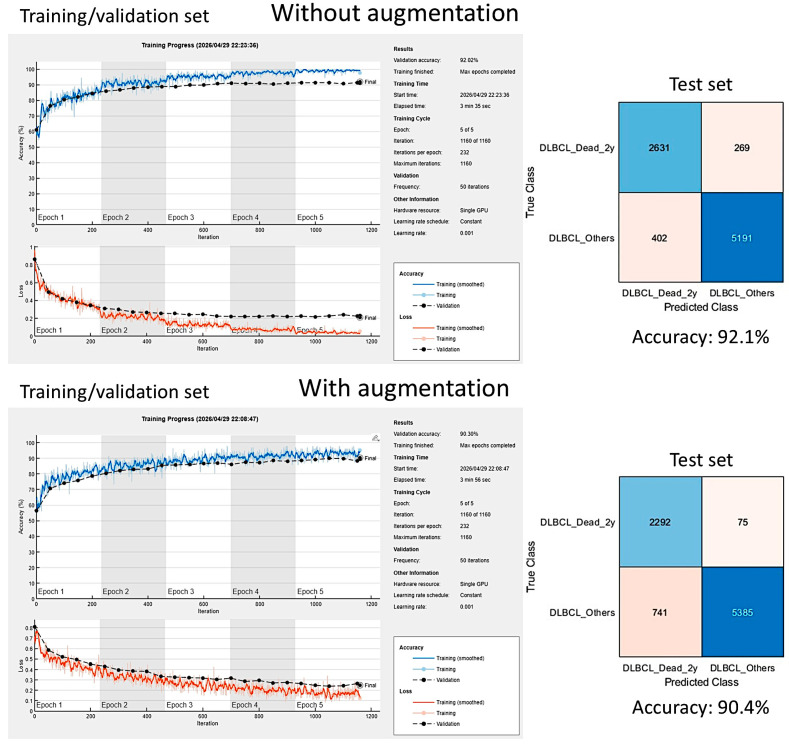
CNN experiment with data augmentation using ResNet18.

First, the series was split into a training set (70%), a validation set (10%), and a test set (20%) to help prevent overfitting during training. The training set patches were shuffled before training. No augmentation options were used during the training, including random reflection axis, rotation (degrees), rescaling, horizontal translation (pixels) or vertical translation (pixels); however, Appendix G (Figure A7) shows CNN with data augmentation. To avoid overfitting, the initial learning rate was set to 0.001, and the maximum number of epochs was set to five.

The training options were as follows: sgdm solver, 0.001 initial learning rate, constant learning rate schedule, 128 minibatch size, 5 max epochs, and a validation frequency of 50. The method is as previously described [57,58,59,60]. In the NasNet-Large CNN, due to hardware limitations to run the analysis, the minibatch size was set at 16. All hyperparameters were consistent and unified between CNNs and are described in Appendix H.

Image patches were classified using transfer learning and 20 different types of CNN, including AlexNet, DarkNet-19, DarkNet-53, DenseNet-201, EfficientNet-b0, GoogleLeNet, GoogleLeNet-places365, Inception-ResNet-v2, Inception-v3, MobileNet-v2, NasNet-Mobile, NasNet-Large, ResNet-18, ResNet-50, ResNet-101, Shufflenet, SqueezeNet, VGG-16, VGG-19, and Xception.

Performance metrics were accuracy (%), precision (%), recall (%), false positive rate (%), specificity (%), and F1 score (%).

The following hardware and software were used: NanoZoomer S360, #C13220-01 (Hamamatsu Photonics K.K.); 12-Core AMD Ryzen 9 5900X CPU, 4900 MHz; 49075 MB DDR4-3200 SDRAM; NVIDIA GeForce RTX 4080 SUPER GPU; MATLAB R2023b Update 10 (23.2.0.2859533), 64-bit (win64); PhotoScape v3.7; IBM SPSS Statistics version 27 (Release 27.0.1.0, 64-bit edition); and NDP.view 2.9.29 (RUO) 2022/01/14 (Hamamatsu Photonics K.K.).

**Figure 2:**
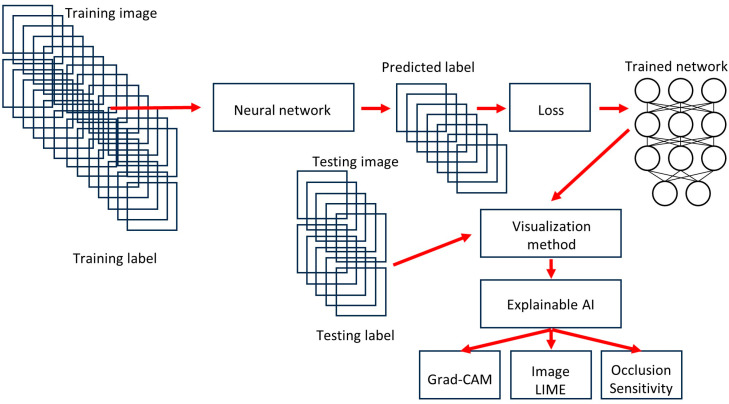
Methodology. The series was randomly split into training (70%) and validation (10%) sets for training, and a test (20%) set using new data. Explainable artificial intelligence (XAI) was performed using Grad-CAM, image LIME, and occlusion sensitivity. First, since the aim was to identify which models were more suitable (best time and performance ratio) and to create a pretrained convolutional neural network (CNN) to be used in the future as transfer learning in a larger series of cases of lymphoma, the initial analysis was patch-based. All image patches of 224 × 224 × 3 were pooled into three different folders: a training set for training the network, a validation set for testing performance during training, and a test set used after training to assess how well the network performed on new data. All patches were mutually exclusive between folders; no repeated patches were found. During the deep learning workflow, common types of transformations, such as geometric transformations, cropping, and adding noise, were not performed. Secondly, all analyses were repeated using a hybrid partitioning and test set at the patient level to avoid information leakage.

**Figure 3:**
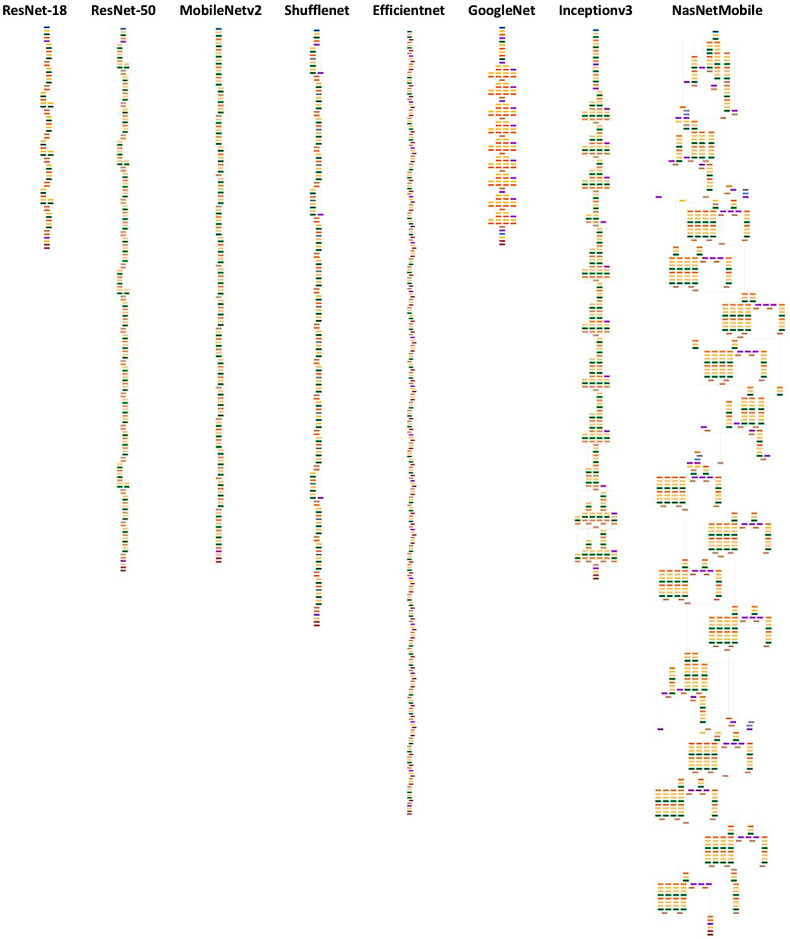
Architectures of the main different types of convolutional neural networks used in this study.

**Figure 4:**
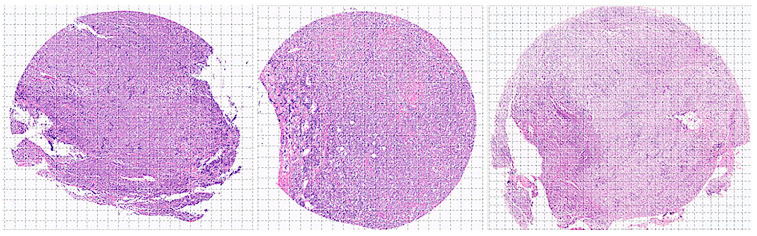
Image splitting. Histological images stained with hematoxylin and eosin (H&E) were first digitized at 200 × magnification and 150 dpi. Later, the images were split at 224 × 224 × 3 resolution, which is suitable for convolutional neural network processing. This image shows the splitting of 3 cases of DLBCL obtained from the H&E of a tissue microarray.

**Figure 5:**
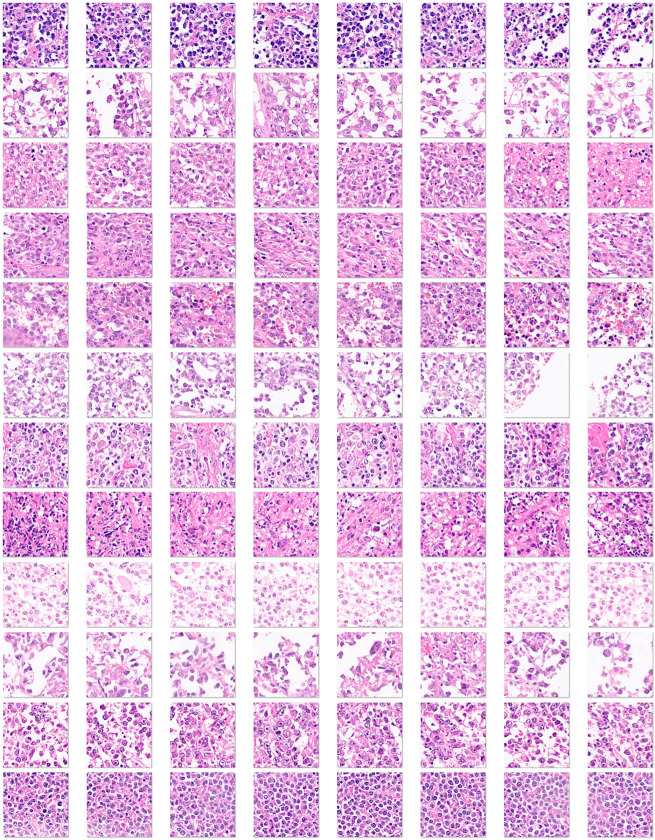
Image splitting of the aggressive group (Dead < 2 years (24 months)). These DLBCL cases were characterized by death events before the first 2 years of overall survival. Histological images stained with hematoxylin and eosin (H&E) were first digitized at 200 × magnification and 150 dpi. Later, the images were split at 224 × 224 × 3 resolution, which is suitable for convolutional neural network processing. This image shows the splitting of 12 cases of DLBCL, which is a heterogeneous clinicopathologic entity. DLBCL is derived from germinal center B cells (centroblasts) or post-germinal activated B cells (immunoblasts).

**Figure 6:**
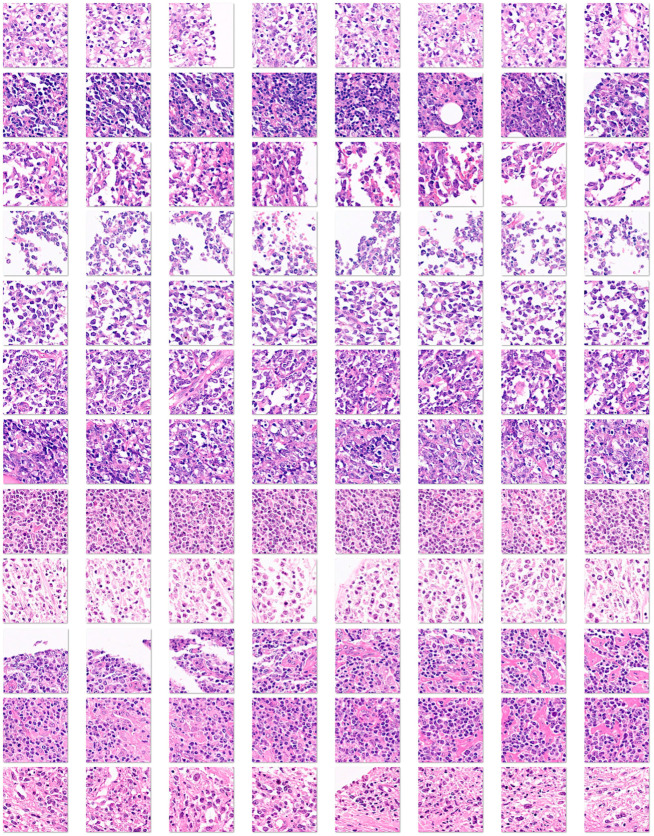
Image splitting of the Others group. This image shows examples of DLBCL cases that did not have a death event before the 2 years of overall survival follow-up (i.e., less aggressive cases, “others”). Histological images stained with hematoxylin and eosin (H&E) were first digitized at 200 × magnification and 150 dpi. Later, the images were split at 224 × 224 × 3 resolution, which is suitable for convolutional neural network processing. This image shows the splitting of 12 cases of DLBCL. DLBCL is a heterogeneous clinicopathologic entity, derived from germinal center B cells (centroblasts) or post-germinal activated B cells (immunoblasts).

**Figure 7:**
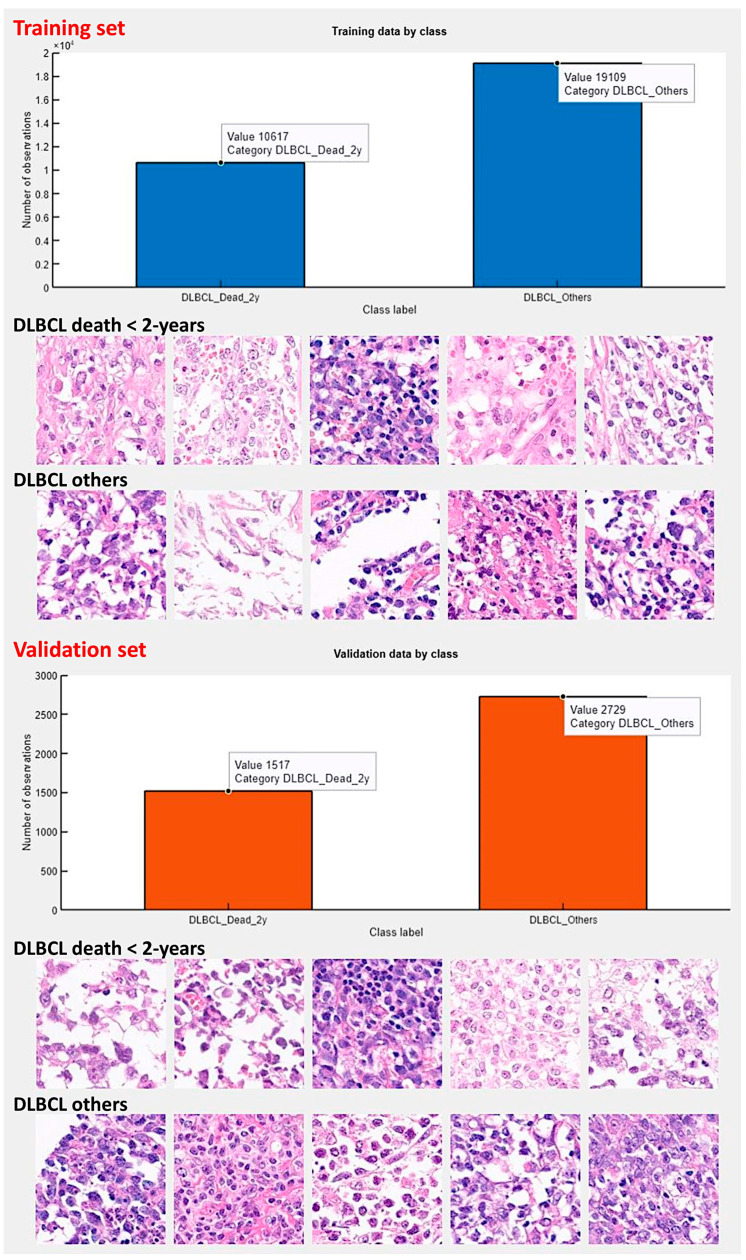
Training and validation datasets. The series was split into a training set (70%), a validation set (10%) to help prevent overfitting, and a test set (20%). No augmentation options were used during the training. This figure shows the number of image patches and random example images from the training and validation sets.

Figure 2, Figure 3, Figure 4, Figure 5, Figure 6 and Figure 7 depict the methodological design.

## 3. Results

The results include several dataset combinations as follows:

- *Analysis of Dataset 1.* Based on the overall survival curve and the 2-year inflection point, 2 survival groups were defined: the aggressive clinical behavior group, characterized by a death event before 2 years (“Dead < 2 years”), and the “Others” group.
- *Alternative Dataset 2:* exclusion of alive cases with follow-up of less than 2 years.
- *Alternative Dataset 2:* exclusion of alive DLBCL cases with a follow-up of less than 2 years, hybrid partitioning to avoid information leakage and patient-based analysis.
- *Alternative Dataset 3*: exclusion of alive DLBCL cases with a follow-up of less than 2 years and addition of the third group of reactive lymphoid tissue.

### 3.1. Analysis of Dataset 1

Based on the overall survival curve and the 2-year inflection point, 2 survival groups were defined: the aggressive clinical behavior group, characterized by a death event before 2 years (24 months) (“Dead < 2 years”), and the “Others” group.

#### 3.1.1. Dataset 1: CNN Performance Analyses and Image Classification

**Figure 8:**
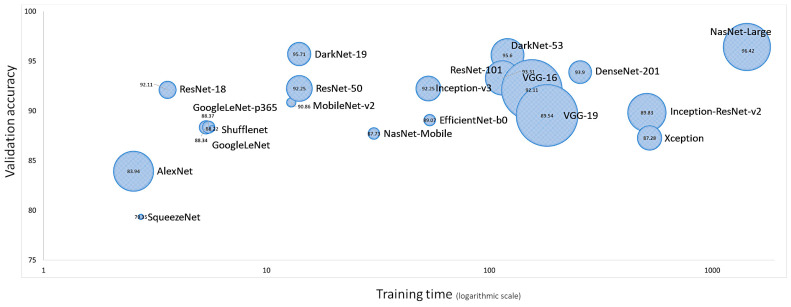
Neural network training performances. The most important characteristics are the neural network accuracy (y-axis), speed (x-axis), and size (circle). Choosing a neural network is a tradeoff between these characteristics. NasNet-Large had the best validation accuracy (96.42%), followed by DarkNet-19 (95.71%). However, in relation to AlexNet, which was the fastest, NasNet-Large took 564.2 times longer to compute (i.e., relative time).

**Figure 9:**
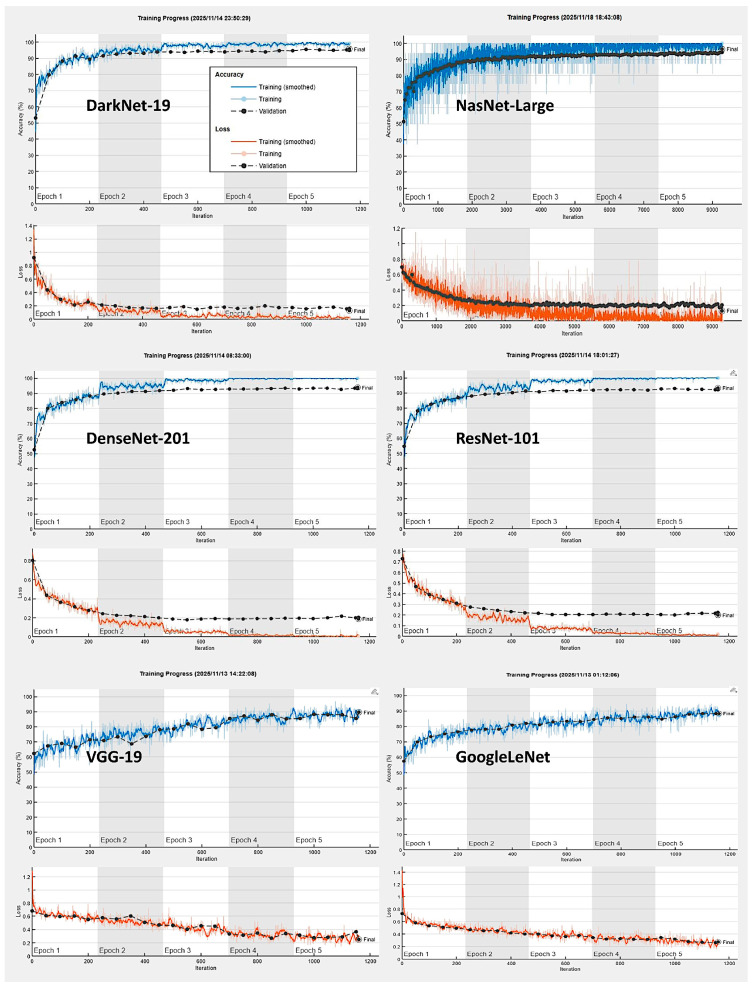
CNN training plots.

In the training/validation set, DarkNet-19 had the best validation accuracy (95.71%), and the accuracy/training time ratio was moderate (0.114). The most efficient architecture was ResNet-18, which had a validation accuracy of 92.11% and a ratio of 0.426 (Table 3 and Figure 8 and Figure 9).

**Figure 10:**
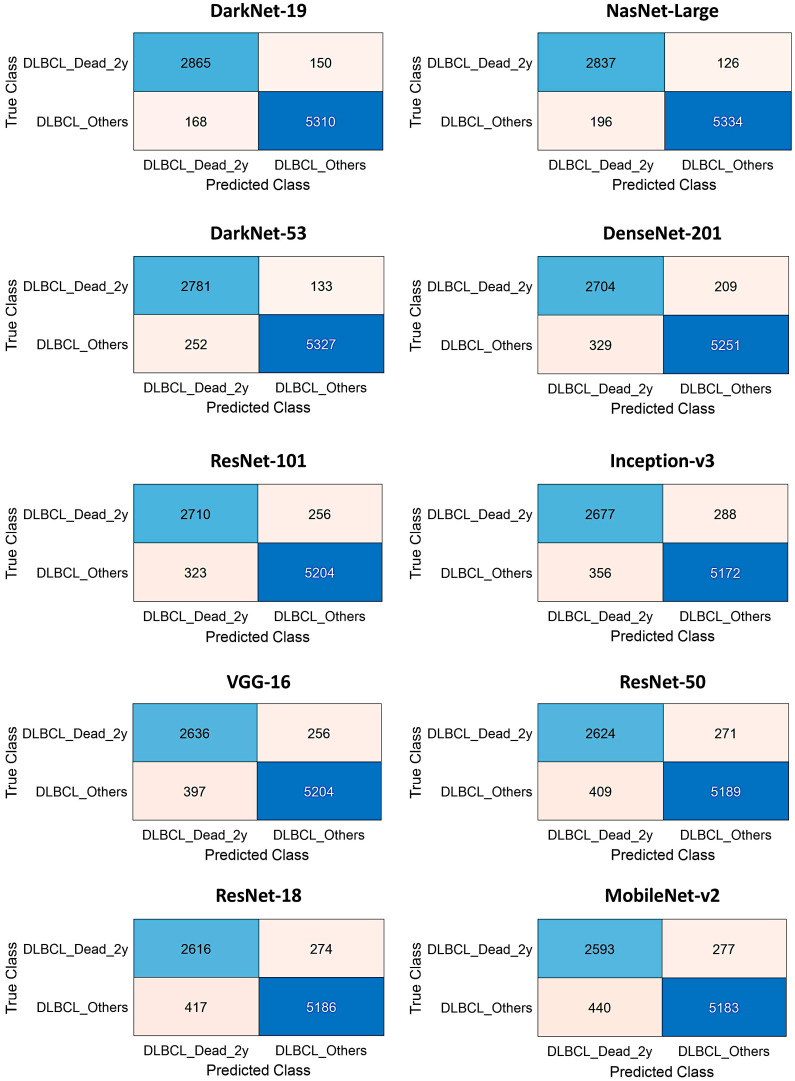
Test set confusion charts.

**Figure 11:**
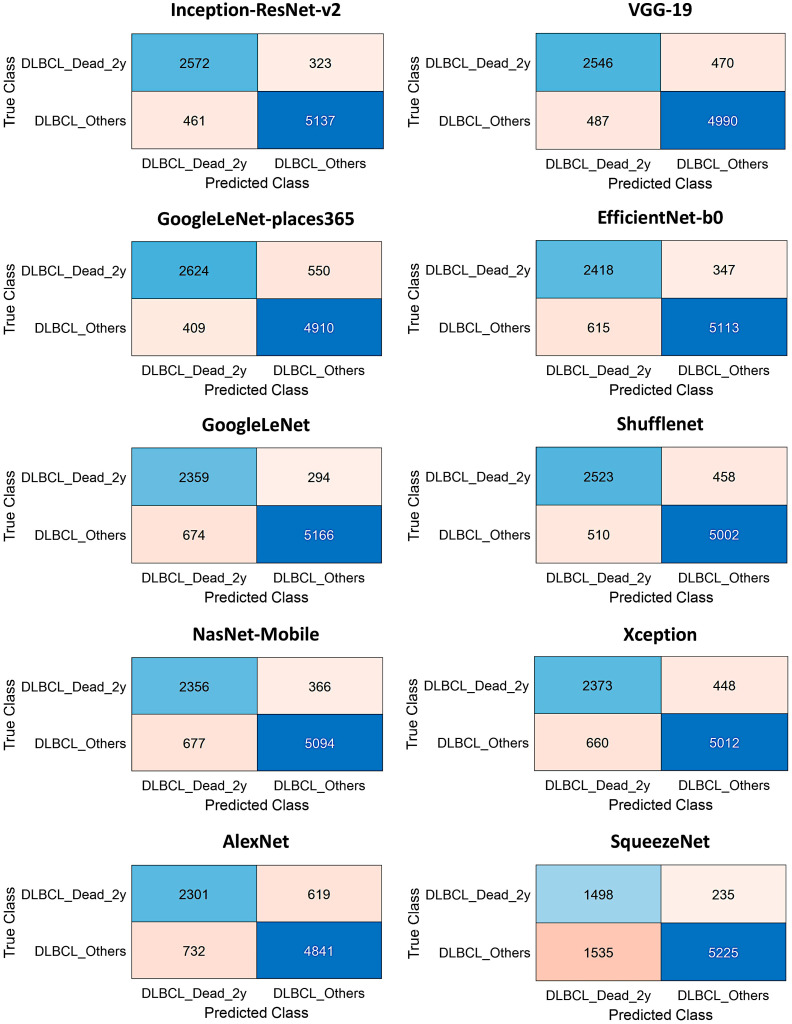
The test set confusion charts (continued).

**Figure 12:**
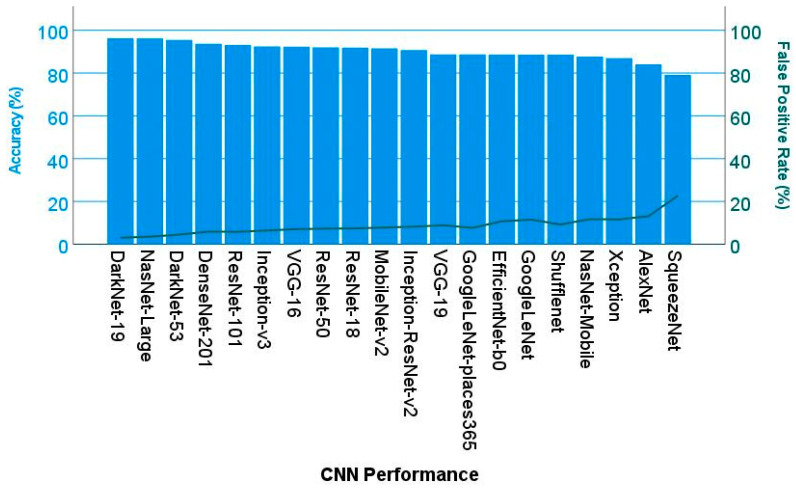
Test set CNN performance. This figure shows the performance parameters of accuracy and the false positive rate of different CNNs.

In the validation set, DarkNet-19 had the best accuracy (96.26%), followed by NasNet-Large (96.21%), DarkNet-53 (95.47%), and DenseNet-201 (93.67%). The confusion charts of the models with higher performance are shown in Figure 10 and Figure 11. All the performance parameters of the models in the test set are shown in Table 4 and Figure 12.

**Figure 13:**
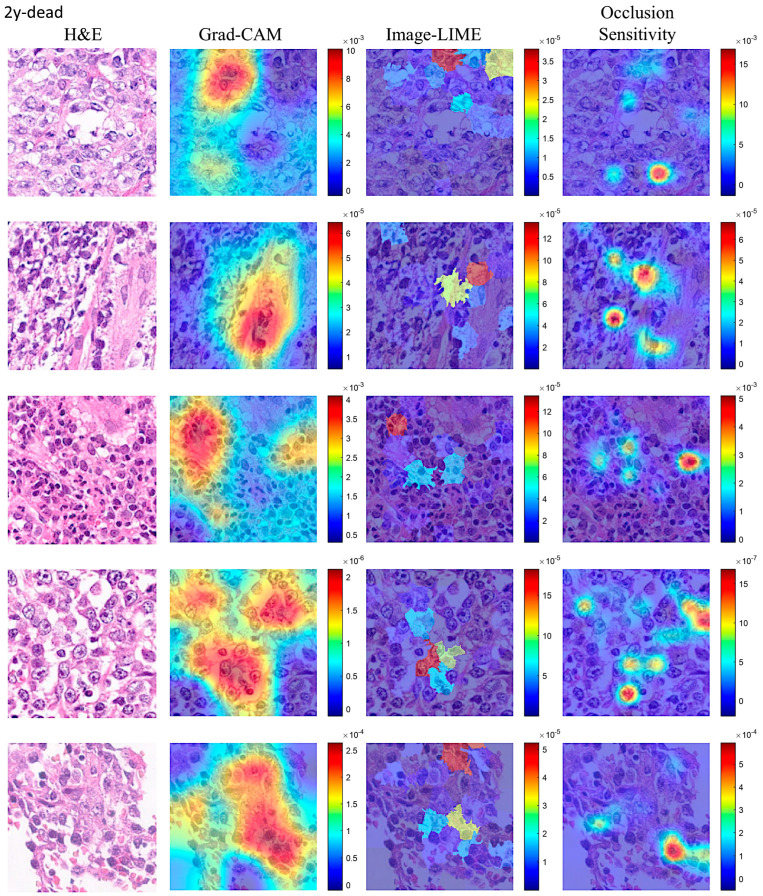
Explainable artificial intelligence (XAI) of the Dead < 2 years (24 months) group. This figure shows 4 different image patches analyzed with XAI techniques, including grad-CAM, image LIME, and occlusion sensitivity. XAI techniques were used to identify the areas of an image that the network DarkNet-19 used for classification. XAI showed that CNNs focused on the epithelial component of neoplasia.

**Figure 14:**
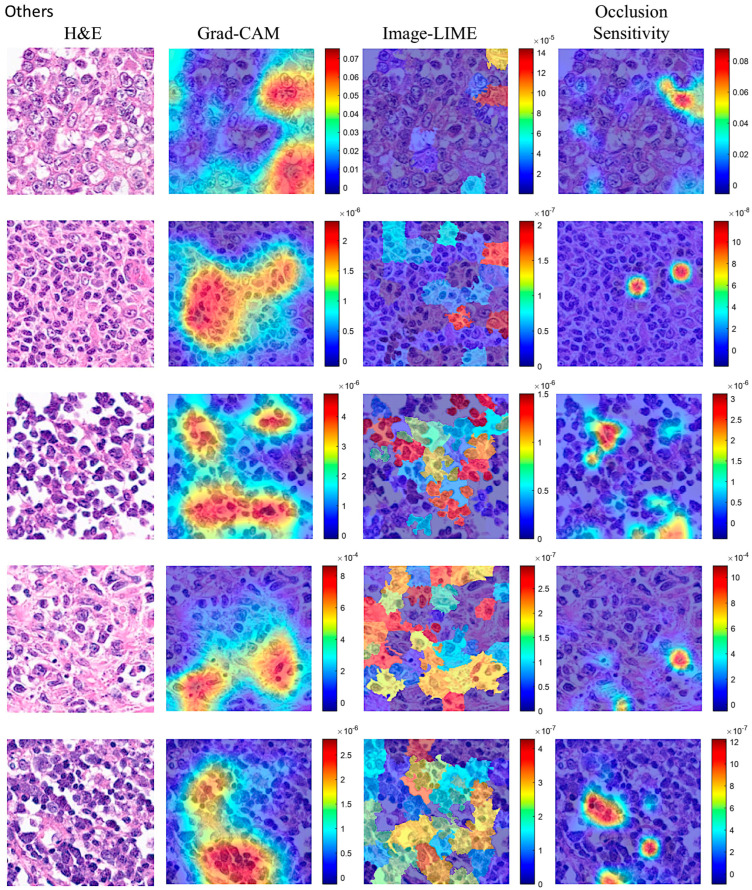
Explainable artificial intelligence (XAI) of the Others group. This figure shows 4 different image patches analyzed with XAI techniques, including Grad-CAM, ImageLIME, and OcclusionSensitivity. XAI techniques were used to identify the areas of an image that the Darknet-19 network used for classification. XAI showed that the convolutional neural networks focused on the epithelial component of the neoplasia.

Explainable artificial intelligence (XAI) was used to identify the areas of the images that the network DarkNet-19 used for classification. Figure 13 and Figure 14 show the Grad-CAM, occlusion sensitivity, and image LIME analysis.

#### 3.1.2. Dataset 1: Clinicopathological Characteristics

**Figure 15:**
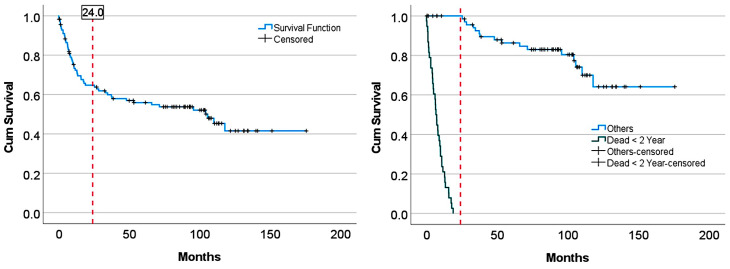
Overall survival of the series. The survival curve showed a point of inflection at 2 years (24 months). Two groups were defined: patients who died before 2 years and others. The others included 8 patients who were alive (censored) before 2 years, and patients who died or were alive after 2 years.

**Figure 16:**
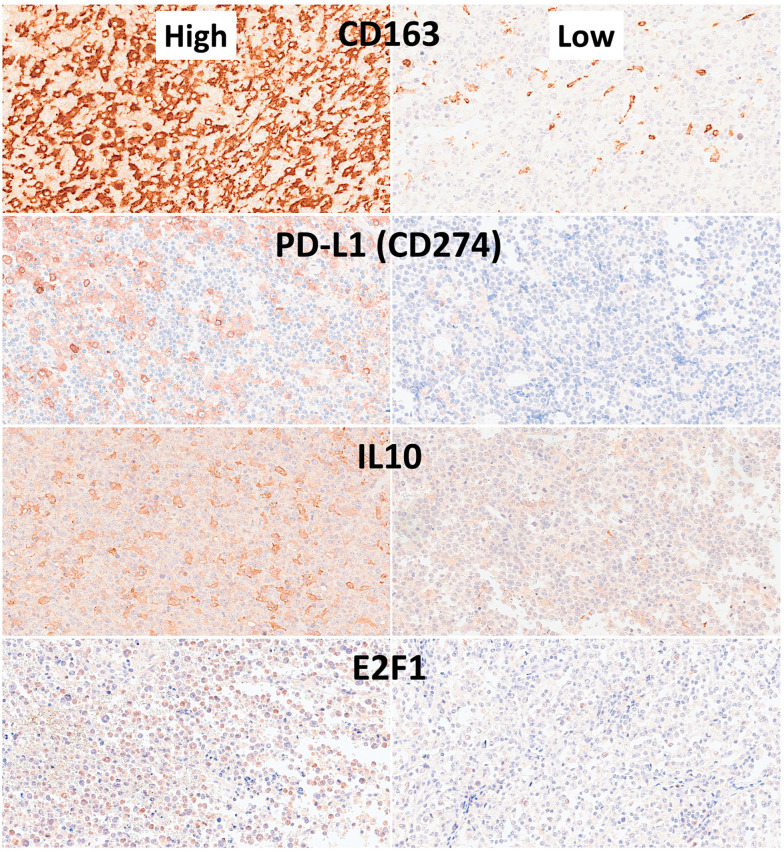
Most relevant immune microenvironment and cell cycle immunohistochemical markers. Analysis of the immune microenvironment, cell cycle, and germinal center markers showed that cases of Dead < 2 years (24 months) had higher CD163, IL10, and PD-L1 levels, but lower E2F1 levels (all *p* values < 0.05).

**Figure 17:**
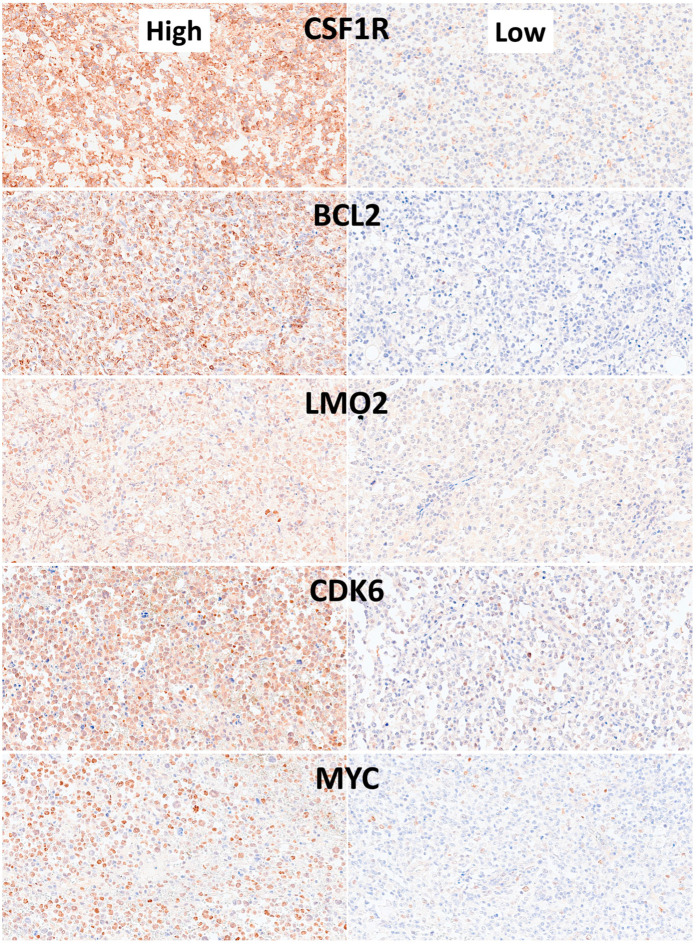
Additional immune microenvironment and cell cycle immunohistochemical markers. For these markers, the analysis of the immune microenvironment, cell cycle, and germinal center markers showed no statistically significant differences at the quantitative level (all *p* values > 0.05).

The Dead < 2 years group was correlated with several clinicopathological characteristics of the patients. The aggressive patients correlated with stage III–IV, International Prognostic Index (IPI) High + High/intermediate, absence of clinical response to treatment, non-GCB cell of origin, CD10−, BCL2+, and EBER+. Analysis of the immune microenvironment, cell cycle, and germinal center markers showed that aggressive patients had higher CD163, IL10, and PD-L1, and lower E2F1 protein expressions (all *p* values < 0.05) (Table 5 and Figure 15, Figure 16 and Figure 17).

### 3.2. Alternative Dataset 2: Exclusion of Alive Cases with Follow-Up of Less than 2 Years

In the original dataset, there were 8 alive cases (censored) with a follow-up of less than 2 years that we included in the “others” group. One may consider that these cases should be excluded from the series. Therefore, the analysis was repeated with these 8 cases excluded using the DarkNet-19 CNN.

In the design of the DarkNet-19 architecture, changes in convolution2dLayer number 19 are necessary. The following parameters were used: name (conv19), filter size (1, 1), number of filters (2), stride (1, 1), dilation factor (1, 1), padding (same), padding value (0), weights [ ], bias [ ], weight learning rate factor (10), weightL2 factor (1), bias learning rate factor (10), biasL2 factor (0), weights initializer (glorot), and bias initializer (zeros). In the last classification layer, the parameter classes and output size are set to (auto).

The training options were as follows: solver (sgdm), initial learning rate (0.001), minibatch size (128), max epochs (5), validation frequency (50), solver momentum (0.9), learning rate schedule (none), drop factor (0.1), and period (10). Normalization and regularization included L2 regularization (0.0001), reset input normalization (yes), and batch normalization statistics (population). Mini-batch shuffle was set to (every-epoch). For validation and output, validation patience was set to (Inf) and output network to (last-iteration). Gradient clipping used gradient threshold method (I2norm), with a gradient threshold of (Inf). The hardware execution environment was set to (auto). Checkpoint path was ( ), frequency (1), and frequency unit (epoch).

**Figure 18:**
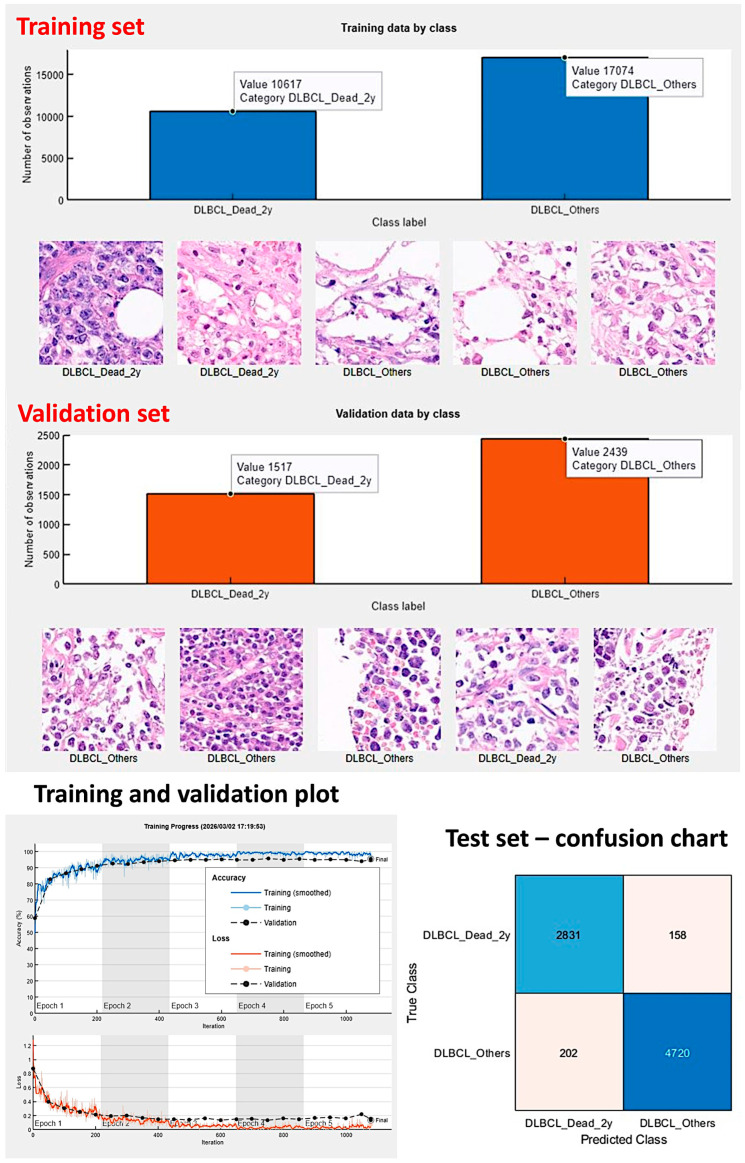
Analysis using modified dataset 1. The analysis was repeated with the exclusion of 8 cases of alive patients who had a follow-up of less than 2 years and were previously included in the “Others” group. This analysis, also based on the patch level because the aim was to create a trained CNN for transfer learning in the future, was performed using the DarkNet-19 architecture. In the test set, the accuracy was 95.5%. Patch-level analysis.

The training elapsed 13 min and 6 s after the 5 epochs were completed. The training cycle comprised 1080 iterations, with 216 iterations per epoch. The validation accuracy was 95.40%. In the testing set, the accuracy was 95.5%. Other performance parameters were precision 93.34%, recall 93.34%, false positive rate 4.1%, specificity 95.9%, and F1 score 93.34% (Figure 18).

**Figure 19:**
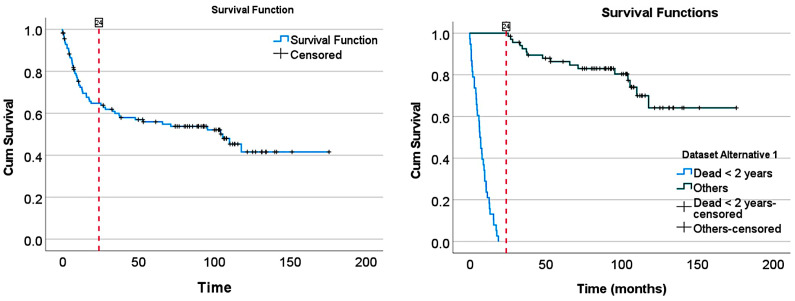
Overall survival of alternative dataset 1. In the original dataset, there were 8 alive cases (censored) with a follow-up of less than 2 years (24 months) that we included in the “Others” group. It may be considered that these cases should be excluded from the series. Therefore, the analysis was repeated with these 8 cases excluded using the DarkNet-19 CNN.

The characteristics of the survival curve are shown in Figure 19.

Statistical analyses were performed to describe the clinicopathological characteristics of the series as well as to identify which variables were different between the 2 groups. In comparison to Others (i.e., Dead or Alive > 2 years), the Dead < 2 years patients were characterized by IPI High/High-Intermediate, absence of clinical response, higher sIL2R, and Epstein–Barr virus (EBER) positivity; the phenotype was CD10 negative, BCL2 positive, and non-GCB according to the cell-of-origin surrogate Hans’classifier; and the immune-oncology markers of high IL10, PD-L1 and CD163, but lower E2F1, levels (Table 6).

### 3.3. Alternative Dataset 2: Exclusion of Alive DLBCL Cases with a Follow-Up of Less than 2 Years and Hybrid Partitioning to Avoid Information Leakage and Patient-Based Analysis

**Figure 20:**
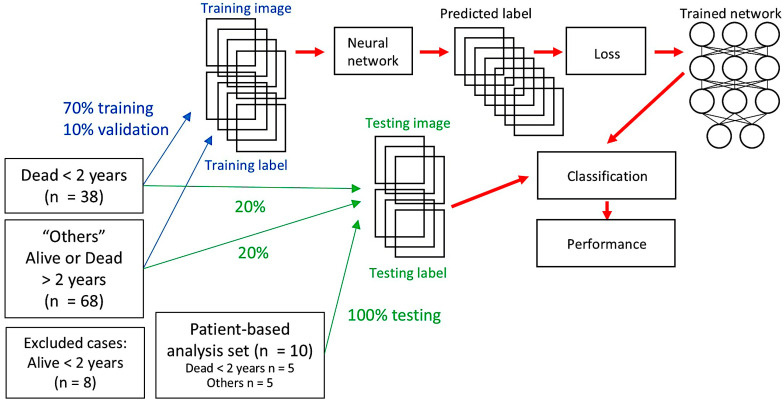
Hybrid partitioning. This hybrid partitioning strategy aimed to prevent information leakage. Initial analysis was patched-based and used 38 cases of Dead < 2 years (24 months) and 68 cases of “Others”. The “Others” group was composed of Alive or Dead > 2 years patients. Eight alive cases < 2 years were excluded from the series. The test set 2 comprised 10 cases, and the analysis was patient-based.

**Figure 21:**
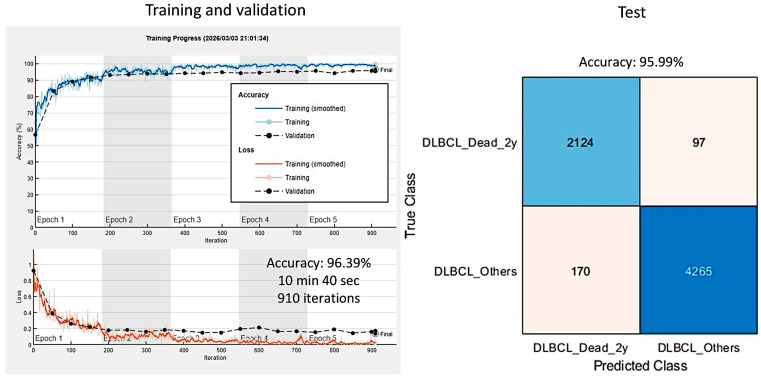
Hybrid partitioning strategy. This figure shows the training/validation and testing of the DarkNet-19 CNN used in hybrid partitioning, with test set 1 being image-patch-based. Ten random cases—5 of Dead < 2 years and 5 of Others—were selected as test set number 2. The training/validation was performed at the patch level. Test set 1 was also performed at the patch level. However, test set 2 was performed at the patient level (Table 7).

Hybrid partitioning was performed to confirm whether information leakage was present in the classification model. Ten random cases—5 of Dead < 2 years and 5 of Others—were selected as test set number 2. The training/validation was performed at the patch level. Test set 1 was also at the patch level. However, test set 2 was performed at the patient level. In the patient-level analysis, ROC analysis was used to determine the best cutoff to differentiate between the 2 groups. In this analysis, the 8 cases of alive DLBCL before 2 years were excluded. Data is shown in Figure 20 and Figure 21 and Table 7.

### 3.4. Alternative Dataset 3: Exclusion of Alive DLBCL Cases with a Follow-Up of Less than 2 Years and Addition of the Third Group of Reactive Lymphoid Tissue

**Figure 22:**
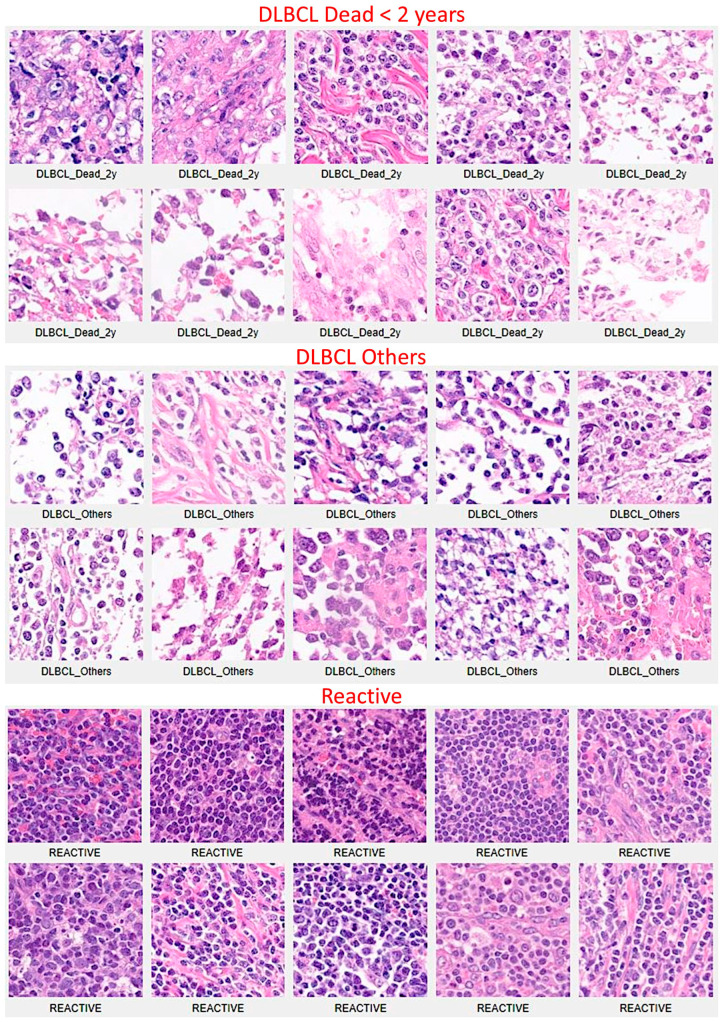
Image patches of DLBCL and reactive lymphoid tissue. The series included 44 cases of reactive lymphoid tissue to expand the classification into three different diagnostic groups: Dead < 2 years, Others, and Reactive lymphoid tissue. In total, 8 cases of Alive < 2 years were excluded from the analysis.

**Figure 23:**
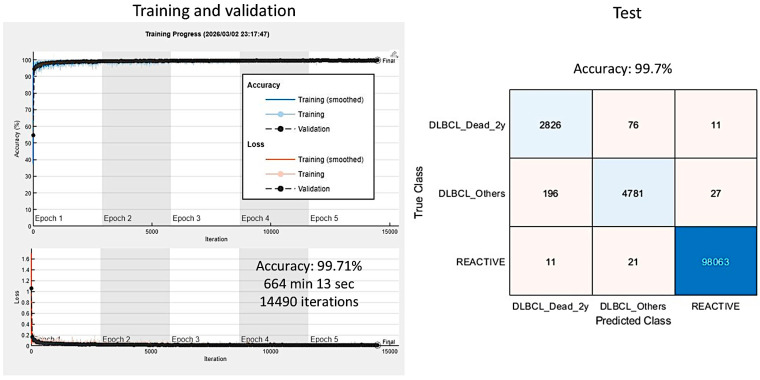
Training progress and test set performance. The accuracy of the training/validation set was 99.71%. It consisted of 14,490 iterations, 5 epochs, and lasted for 664 min and 13 s. In the test set, the accuracy was 99.7%. Patch-level analysis was performed to classify between DLBCL dead < 2 years, DLBCL Others, and reactive lymphoid tissue.

Classification with reactive lymphoid tissue is also important for differential diagnosis. Therefore, an updated series included 44 reactive lymphoid tissue cases, 38 DLBCL cases in the group “Dead < 2 years”, and 68 DLBCL cases in the group “Others” (which included both Dead and Alive > 2 years) (Figure 22). Of note, 8 cases of Alive < 2 years were excluded as mentioned in Section 3.3. At the patch level, the accuracy was 99.7% (Figure 23).

Further analysis was performed to train the network to classify between DLBCL and reactive lymphoid tissue using a hybrid partitioning. Ten aleatory cases of DLBCL and 5 cases of reactive lymphoid tissue were excluded from the main series and analyzed at patient level as test set 2. The rest of the cases were analyzed at the patch level, and the series was split into a training set (70%), a validation set (10%) to help prevent over-fitting during training, and test set 1 (20%).

**Figure 24:**
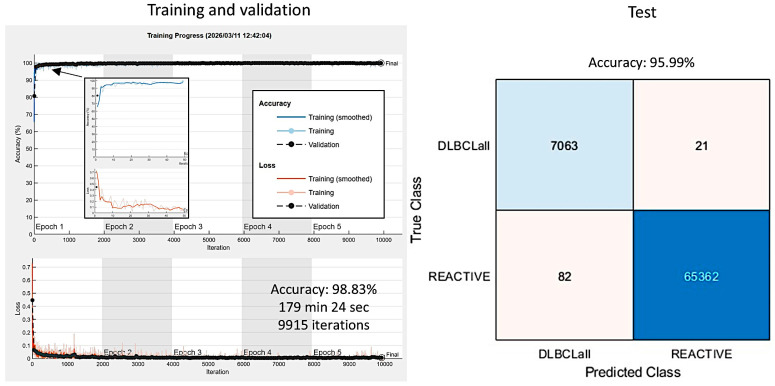
Training progress and test set performance. The accuracy of the training/validation set was 98.83%. It consisted of 9915 iterations, 5 epochs, and lasted for 179 min and 24 s. In the test set, the accuracy was 95.99%. Patch-level analysis was performed to classify between DLBCL and reactive lymphoid tissue.

The training took 179 min and 24 s. The training cycle included 5 epochs, with 1983 iterations per epoch, and a total of 9915 iterations. The validation accuracy was 99.83%. In test set 1, the accuracy was 95.99%. This analysis was patch-based (Figure 24).

The test set 2 analysis was performed at the patient level, including 10 cases of DLBCL and 5 cases of reactive lymphoid tissue. All cases but one (90%) of the DLBCL cases were correctly classified as DLBCL. All cases of reactive lymphoid tissue were correctly classified (100%). This analysis was designed to avoid information leakage (Table 8).

Finally analyses of groups Dead < 2 years and Dead > 2 years and hyperparameter tuning are shown in Appendix C and Appendix D. Appendix E shows hyperparameter tuning in DLBCL vs. reactive lymphoid tissue. Appendix F shows the architecture of DarkNet-19.

## 4. Discussion

DLBCL is one of the most common diagnostic categories of non-Hodgkin lymphoma (NHL), accounting for approximately 25% of NHL cases in the developed world [2,5]. This study focused on one of the most frequent lymphomas. Therefore, it is of interest to the medical community.

Histologically, DLBCL is characterized by large transformed B cells that depict a diffuse growth pattern and efface the normal architecture of the underlying histological structure [2,5]. The diagnostic category of DLBCL, NOS, refers to conventional DLBCL with a mature B-cell phenotype and large cell morphology, but lacking none of the criteria that define specific large B-cell lymphoma subtypes (i.e., other large B-cell variants) [2,5,25].

This study used a series of 114 cases of conventional large B-cell lymphoma. Overall, it included not only NOS cases but also 28 cases of EBER-positive DLBCL. Most of the cases were nodal (58/114, 50.9%), followed by other extranodal (28.1%) and gastrointestinal (11.4%). The aim of the project was to identify cases with poor prognosis using only H&E staining and a CNN. After the evaluation of the overall survival curve, 2 groups were defined: patients who died before 2 years and others. Correlation with the clinicopathological characteristics found that the <2 years group was correlated with stage III–IV, International Prognostic Index (IPI) High + High/intermediate, progressive disease, non-GCB cell of origin, CD10−, BCL2+, and EBER+. Analysis of the immune microenvironment, cell cycle, and germinal center markers showed that the Dead < 2 years group had higher IL10, CD163, PD-L1 levels, but lower E2F1 expression. Therefore, the CNN managed to identify the histological features that correlated with the prognosis of the patients—features that were not very obvious under conventional histological examination with an optical microscope. Notably, our study only analyzed H&E stainings, and the immunohistochemical data was used to evaluate the differences between the two groups. However, in a future study, the immunohistochemical images could be included in the experimental design.

As described in the Materials and Methods section, the deep learning workflow includes preprocessing data, importing and building the network, training the network, tuning the network, and visualizing the results. In this study, we used transfer learning to take advantage of the knowledge provided by a pretrained network to learn new patterns in new data.

Fine-tuning a pretrained network with transfer learning is typically much faster and easier than training from the beginning. The reuse of the pretrained network includes the following steps: load the pretrained network, replace the final layers, train the network, predict and assess the network accuracy, and deploy the results. This study used 20 pretrained networks that are among the best CNNs for image classification, namely AlexNet, DarkNet-19, DarkNet-53, DenseNet-201, EfficientNet-b0, GoogleLeNet, GoogleLeNet-places365, Inception-ResNet-v2, Inception-v3, MobileNet-v2, NasNet-Large, NasNet-Mobile, ResNet-101, ResNet-18, ResNet-50, Shufflenet, SqueezeNet, VGG-16, VGG-19, and Xception. The CNN architectures were different, and the performance ranged from 79.16% for SqueezeNet to 96.26% for DarkNet-19. The training times differed as well, being 1429 min for NasNet-Large and 2 min for AlexNet. The final analysis was performed using DarkNet-19. The final model based on DarkNet-19 predicted prognosis groups with high performance (test set accuracy = 96.26%). The other performance parameters were precision (94.46%), recall (95.02%), false positive rate (3.07%), specificity (96.93%), and F1 score (94.74%). Our data indicates that CNNs can manage to identify histological characteristics of the samples that are difficult to evaluate by pathologists. This use of narrow artificial intelligence in the field of histopathology may be useful in the future.

A machine learning model is often referred to as a “black box” model because understanding how the model makes predictions can be difficult. Interpretability tools help overcome this aspect of machine learning algorithms and reveal how predictors contribute (or do not contribute) to predictions. Moreover, you can validate whether the model uses the correct evidence for its predictions and find model biases that are not immediately apparent. In this study, explainable artificial intelligence (XAI) was performed using Grad-CAM, occlusion sensitivity and image LIME on the network DarkNet-19. Overall, XAI showed that the CNNs focused on the neoplastic B lymphocytes, but some components of the microenvironment may also have had an influence, as shown by the high infiltration of tumor-associated macrophages in the Dead < 2 years group. Of note, current XAI techniques do not allow for a more detailed analysis of this type of histological tissue.

If images are affected by noise, this may lead to biases in the final results; enhancement and restoration techniques may be desirable [61,62].

A limitation of this study is the small sample size, which consists of 114 cases. However, the immunohistochemical analysis was comprehensive. In the future, larger series of cases and the inclusion of immunohistochemical images into the CNN may improve the experimental design. Because of the heterogeneity of DLBCL, a series of around 300 cases is desirable.

## 5. Conclusions

In conclusion, narrow artificial intelligence (i.e., AI trained to perform a specific or a set of closely related tasks) can predict the prognosis of DLBCL based on the computer vision CNN histological analysis of H&E images, but it is process-specific and operates within limited constraints.

## Figures and Tables

**Table 1:** Difference in death event between the two prognostic groups, curve estimation.

Model Summary					Parameter Estimates		
Equation	R Square	F	*p* value	Constant	B1	B2	B3
Linear	0.825	528.5	<0.001	0.831	−0.003		
Quadratic	0.893	461.205	<0.001	0.874	−0.007	2.565 × 10^−5^	
Cubic	0.940	572.406	<0.001	0.918	−0.013	0.000	−4.412 × 10^−7^
Compound	0.888	888.351	<0.001	0.833	0.995		
Growth	0.888	888.351	<0.001	−0.183	−0.005		
Exponential	0.888	888.351	<0.001	0.833	−0.005		

**Table 2:** Difference in death event between the two prognostic groups.

	Alive	Dead	*p* Value	Exp (B)	95% C.I. for Exp (B)
“Aggressive”	52/68 (76.5%)	16/68 (23.5%)	<0.001	15.4	5.9–39.8
“Moderate”	8/46 (17.4%)	38/46 (82.6%)	<0.001	0.31	

C.I., confidence internal.

**Table 3:** Training/validation set CNN performance.

CNN Model	Learnables	Layers	Connections	Image Input	Training Time	Validation Accuracy (%)	Efficiency	Relative Time
NasNet-Large	84.9 M	1243	1462	331 × 331 × 3	1429 min 12 s	96.42	0.001	564.2
DarkNet-19	20.8 M	64	63	256 × 256 × 3	14 min 1 s	95.71	0.114	5.5
DarkNet-53	41.6 M	184	206	256 × 256 × 3	120 min 35 s	95.6	0.013	47.6
DenseNet-201	20 M	708	805	224 × 224 × 3	255 min 9 s	93.9	0.006	100.7
ResNet-101	44.6 M	347	379	224 × 224 × 3	114 min 21 s	93.31	0.014	45.1
Inception-v3	23.8 M	315	349	299 × 299 × 3	53 min 15 s	92.25	0.029	21.0
ResNet-50	25.5 M	177	192	224 × 224 × 3	14 min 3 s	92.25	0.109	5.5
ResNet-18	11.6 M	71	78	224 × 224 × 3	3 min 36 s	92.11	0.426	1.4
VGG-16	138.3 M	41	40	224 × 224 × 3	155 min 10 s	92.11	0.009	61.3
MobileNet-v2	3.5 M	154	163	224 × 224 × 3	12 min 55 s	90.86	0.117	5.1
Inception-ResNet-v2	55.8 M	824	921	299 × 299 × 3	509 min 8 s	89.83	0.003	201.0
VGG-19	143.6 M	47	46	224 × 224 × 3	181 min 17 s	89.54	0.008	71.6
EfficientNet-b0	5.3 M	290	363	224 × 224 × 3	54 min 0 s	89.07	0.075	21.3
GoogleLeNet-places365	5.9 M	144	170	224 × 224 × 3	5 min 30 s	88.37	0.268	2.2
GoogleLeNet	6.9 M	144	170	224 × 224 × 3	5 min 21 s	88.34	0.275	2.1
Shufflenet	1.4 M	172	187	224 × 224 × 3	5 min 42 s	88.22	0.258	2.3
NasNet-Mobile	5.3 M	913	1072	224 × 224 × 3	30 min 19 s	87.73	0.048	12.0
Xception	22.9 M	170	181	299 × 299 × 3	522 min 52 s	87.28	0.003	206.4
AlexNet	60.9 M	25	24	227 × 227 × 3	2 min 32 s	83.94	0.552	1.0
SqueezeNet	1.2 M	68	75	227 × 227 × 3	2 min 44 s	79.35	0.484	1.1

Efficiency: Validation accuracy/time (%/s). Relative time: faster CNN (AlexNet) as reference.

**Table 4:** The test set CNN performance parameters.

CNN	Accuracy (%)	Precision (%)	Recall (%)	False Positive Rate (%)	Specificity (%)	F1 Score (%)
DarkNet-19	96.26	94.46	95.02	3.07	96.93	94.74
NasNet-Large	96.21	93.54	95.75	3.54	96.46	94.63
DarkNet-53	95.47	91.69	95.44	4.52	95.48	93.53
DenseNet-201	93.67	89.15	92.83	5.9	94.1	90.95
ResNet-101	93.18	89.35	91.37	5.84	94.16	90.35
Inception-v3	92.42	88.26	90.29	6.44	93.56	89.26
VGG-16	92.31	86.91	91.15	7.09	92.91	88.98
ResNet-50	91.99	86.52	90.64	7.31	92.69	88.53
ResNet-18	91.86	86.25	90.52	7.44	92.56	88.33
MobileNet-v2	91.56	85.49	90.35	7.83	92.17	87.85
Inception-ResNet-v2	90.77	84.8	88.84	8.24	91.76	86.77
VGG-19	88.73	83.94	84.42	8.89	91.11	84.18
GoogleLeNet-places365	88.71	86.52	82.67	7.69	92.31	84.55
EfficientNet-b0	88.67	79.72	87.45	10.74	89.26	83.41
GoogleLeNet	88.6	77.78	88.92	11.54	88.46	82.98
Shufflenet	88.6	83.18	84.64	9.25	90.75	83.9
NasNet-Mobile	87.72	77.68	86.55	11.73	88.27	81.88
Xception	86.95	78.23	84.11	11.64	88.36	81.07
AlexNet	84.09	75.87	78.8	13.13	86.87	77.31
SqueezeNet	79.16	49.39	86.44	22.71	77.29	62.86

Recall equals sensitivity and true positive rate. False positive rate equals 1-specificity.

**Table 5:** Correlation with the clinicopathological characteristics.

Variable	All Cases	Dead < 2 Years (24 months)	Others	*p* Value
Frequency	114	38/114 (33.3%)	76/114 (66.7%)	N/A
Clinical characteristics				
Age > 60 years	81/114 (71.1%)	30/38 (78.9%)	51/76 (67.1%)	0.273
Male	60/114 (52.6%)	19/38 (50%)	41/76 (53.9%)	0.697
Location				
Nodal (+Spleen)	58/114 (50.9%)	16/38 (42.1%)	42/76 (55.3%)	0.430
Waldeyer’s ring	11/114 (9.6%)	3/38 (7.9%)	8/76 (10.5%)	
Gastrointestinal	13/114 (11.4%)	5/38 (13.2%)	8/76 (10.5%)	
Other extranodal	32/114 (28.1%)	14/38 (36.8%)	18/76 (23.7%)	
Stage III–IV	46/97 (47.4%)	18/28 (64.3%)	28/69 (40.6%)	0.044
IPI High + High/Intermediate	31/91 (34.1%)	14/27 (51.9%)	17/64 (26.6%)	0.029
RCHOP/RCHOP-like treatment	93/98 (94.9%)	26/28 (92.9%)	67/70 (95.7%)	0.513
Clinical response	68/92 (73.9%)	5/24 (20.8%)	63/68 (92.5%)	<0.001
Hight sIL2R	79/99 (79.8%)	27/29 (93.1%)	52/70 (74.3%)	0.052
Pathological characteristics				
CD3+	0/114 (0%)	0/38 (0%)	0/76 (0%)	1.0
CD20+	114/114 (100%)	38/38 (100%)	76/76 (100%)	1.0
CD5+	13/113 (11.5%)	4/38 (10.5%)	9/75 (12.0%)	1.0
CD10+	33/113 (29.2%)	2/38 (5.3%)	31/75 (41.3%)	<0.001
BCL6+	76/113 (67.3%)	26/38 (68.4%)	50/75 (66.7%)	1.0
MUM1+	93/113 (82.3%)	33/38 (86.8%)	60/75 (80%)	0.442
Non-GCB	77/114 (67.5%)	35/38 (92.1%)	42/76 (55.3%)	<0.001
BCL2+	89/113 (78.8%)	36/38 (94.7%)	53/75 (70.7%)	0.003
*MYC* rearrangement	9/98 (9.2%)	2/29 (6.9%)	7/69 (10.1%)	1.0
EBER+	28/114 (25%)	15/37 (40.5%)	13/75 (17.3%)	0.011
Ki67	16.1% ± 14.2	15.3% ± 12.2	16.5% ± 14.9	0.959
Immune microenvironment				
IL10	12.2% ± 15.8 (n = 102)	18.6% ± 19.6	9.2% ± 12.8	0.006
PD-L1 (CD274)	12.2% ± 15.8% (n = 102)	18.5% ± 19.6	9.1% ± 12.8	0.026
CSF1R	33.5% ± 27.5 (n = 94)	28.7% ± 25.4	35.8% ± 28.3	0.247
CD163	39.2% ± 25.9 (n = 114)	48.2% ± 24.5	34.6% ± 25.6	0.008
CASP8	6.7% ± 8.4 (n = 94)	6.0% ± 9.4	7.1% ± 8.0	0.268
TNFAIP8	41.3% ± 25.6 (n =93)	46.2 ± 24.0	39.3% ± 26.1	0.223
Cell cycle/GC-related				
LMO2	2.6% ± 3.5 (n = 92)	2.4% ± 3.9	2.7% ± 3.4	0.051
MYC	5.4% ± 5.7 (n = 93)	6.5% ± 6.4	4.9% ± 5.5	0.318
MDM2	10.8% ± 8.1 (n = 93)	9.7% ± 6.1	11.3% ± 8.8	0.594
CDK6	5.1% ± 7.4 (n = 93)	3.6% ± 5.3	5.7% ± 8.1	0.056
E2F1	1.8% ± 1.8 (n = 93)	1.2% ± 0.9	2.0% ± 1.9	0.020
BCL2	6.8% ± 9.7 (n = 93)	3.4% ± 4.5	8.1% ± 10.9	0.087
TP53	5.2% ± 8.1 (n = 94)	6.6% ± 10.3	4.6% ± 7.0	0.128

In this series of 114 cases, 8 cases of Alive < 2 years (24 months) were included in the Others group.

**Table 6:** Correlation with the clinicopathological characteristics (Alive < 2 years excluded).

Variable	All Cases	Dead < 2 Years (24 months)	Others (Dead or Alive > 2 Years)	*p* Value
Frequency	106	38/106 (35.8%)	68/106 (64.2%)	N/A
**Clinical characteristics**				
Age > 60 years	75/106 (70.8%)	30/38 (78.9%)	45/68 (66.2%)	0.188
Male	56/106 (52.8%)	19/38 (50%)	37/68 (54.4%)	0.689
Location				
Nodal (+Spleen)	54/106 (50.9%)	16/38 (42.1%)	38/68 (55.9%)	0.430
Waldeyer’s ring	10/106 (9.4%)	3/38 (7.9%)	7/68 (10.3%)	
Gastrointestinal	12/106 (11.3%)	5/38 (13.2%)	7/68 (10.3%)	
Other extranodal	30/106 (28.3%)	14/38 (36.8%)	16/68 (23.5%)	
Stage III–IV	44/90 (48.9%)	18/28 (64.3%)	26/62 (41.9%)	0.069
IPI High + High/Intermediate	30/85 (35.3%)	14/27 (51.9%)	16/58 (27.6%)	0.050
RCHOP/RCHOP-like treatment	88/92 (95.6%)	26/28 (92.9%)	62/64 (96.9%)	0.597
Clinical response	65/87 (74.7%)	5/24 (20.8%)	60/63 (95.2%)	<0.001
Hight sIL2R	75/93 (80.6%)	27/29 (93.1%)	48/64 (75%)	0.049
**Pathological characteristics**				
CD3+	0/106 (0%)	0/38 (0%)	0/68 (0%)	1.0
CD20+	106/106 (100%)	38/38 (100%)	68/68 (100%)	1.0
CD5+	13/105 (12.4%)	4/38 (10.5%)	9/67 (13.4%)	0.766
CD10+	29/105 (27.6%)	2/38 (5.3%)	27/67 (40.3%)	<0.001
BCL6+	70/105 (66.7%)	26/38 (68.4%)	44/67 (65.7%)	0.832
MUM1+	85/105 (81.0%)			
Non-GCB	73/104 (70.2%)	35/38 (92.1%)	38/66 (57.6%)	<0.001
BCL2+	84/105 (80.0%)	36/38 (94.7%)	48/67 (71.6%)	0.005
*MYC* rearrangement	9/92 (9.8%)			
EBER+	25/104 (24.0%)	15/37 (40.5%)	10/67 (14.9%)	0.007
Ki67	15.9% ± 14.5	14.9% ± 12.3	16.4% ± 15.6	0.935
Immune microenvironment				
IL10	9.7% ± 12.2	14.2% ± 14.7	7.3% ± 9.9	0.010
PD-L1 (CD274)	12.3% ± 15.9	18.5% ± 19.6	8.9% ± 12.5	0.027
CSF1R	33.7% ± 27.0	28.1% ± 25.1	36.4% ± 27.7	0.220
CD163	39.9% ± 25.9	48.2% ± 24.5	35.4% ± 25.7	0.014
CASP8	7.1% ± 8.6	5.8% ± 9.2	7.6% ± 8.3	0.139
TNFAIP8	42.3% ± 25.9	46.2% ± 24.1	40.5% ± 26.8	0.336
Cell cycle/GC-related				
LMO2	2.6% ± 3.6%	2.3% ± 3.9	2.7% ± 3.4	0.063
MYC	5.4% ± 5.8	6.3% ± 6.4	4.9% ± 5.5	0.304
MDM2	10.9% ± 8.2	9.4% ± 6.2	11.5% ± 8.9	0.542
CDK6	4.7% ± 6.8	3.5% ± 5.3	5.2% ± 7.4	0.088
E2F1	1.8% ± 1.8	1.1% ± 0.9	2.1% ± 2.0	0.014
BCL2	7.1% ± 9.9	3.5% ± 4.4	8.8% ± 11.2	0.037
TP53	5.4% ± 8.4	6.6% ± 10.3	4.8% ± 7.4	0.108

In this series, the 8 cases of Alive < 2 years were excluded.

**Table 7:** Patient-level analysis in hybrid partitioning.

Case	1	2	3	4	5	6	7	8	9	10
Original diagnosis	Others	Others	Others	Others	Others	D2Y	D2Y	D2Y	D2Y	D2Y
AI-predicted diagnosis 50%	Others	Others	D2Y	Others	Others	D2Y	Others	Others	D2Y	D2Y
AI-predicted diagnosis 77%	Others	Others	D2Y	Others	Others	D2Y	D2Y	Others	D2Y	D2Y
Others’ patches	380	352	62	804	681	15	905	844	74	315
Others’ patches %	94.3	93.9	21.8	96.6	99.4	6.7	60.2	96.6	19.9	43.6
Dead 2 < years (D2Y) patches	23	23	222	28	4	209	599	30	297	408
Dead 2 < years (D2Y) patches %	5.7	6.1	78.2	2.4	0.6	93.3	39.8	3.43	80.1	56.4
All patches	403	375	284	832	685	224	1504	874	371	723
All patches %	100	100	100	100	100	100	100	100	100	100

The standard cutoff for group classification is 50%. By ROC analysis, the best cutoff to differentiate between the 2 groups was 77%. D2Y, Dead < 2 years (24 months). Others, Dead or Alive > 2 years.

**Table 8:** Patient-level analysis in hybrid partitioning including DLBCL and reactive lymphoid tissue.

**Case**	**D1**	**D2**	**D3**	**D4**	**D5**	**D6**	**D7**	**D8**
Original diagnosis	DLBCL	DLBCL	DLBCL	DLBCL	DLBCL	DLBCL	DLBCL	DLBCL
AI-predicted diagnosis	DLBCL	DLBCL	DLBCL	DLBCL	DLBCL	DLBCL	DLBCL	Reactive
DLBCL (D) patches	208	710	1498	305	472	284	719	303
DLBCL (D) patches %	98.6%	96.3%	99.6%	91.3%	79.6%	100%	85.7%	41.9%
Reactive (R) patches	3	27	6	29	121	0	120	420
Reactive (R) patches %	1.4%	3.7%	0.4%	8.7%	20.4%	0%	14.3%	58.1%
All patches	211	737	1504	334	593	284	839	723
All patches %	100	100	100	100	100	100	100	100
**Case**	**D9**	**D10**	**R1**	**R2**	**R3**	**R4**	**R5**	
Original diagnosis	DLBCL	DLBCL	Reactive	Reactive	Reactive	Reactive	Reactive	
AI-predicted diagnosis	DLBCL	DLBCL	Reactive	Reactive	Reactive	Reactive	Reactive	
DLBCL (D) patches	678	546	7	15	2	3	55	
DLBCL (D) patches %	98.9%	66.0%	0.1%	0.03%	0.01%	0.007%	2.59%	
Reactive (R) patches	7	281	38720	59728	17157	45834	2069	
Reactive (R) patches %	1.1%	34.0%	99.9%	99.97	99.99%	99.993%	97.41%	
All patches	685	827	38727	59743	17159	45837	2124	
All patches %	100	100	100	100	100	100	100	

The standard cutoff for group classification is 50%. D, DLBCL. Reactive, reactive lymphoid tissue.

**Figure A1:**
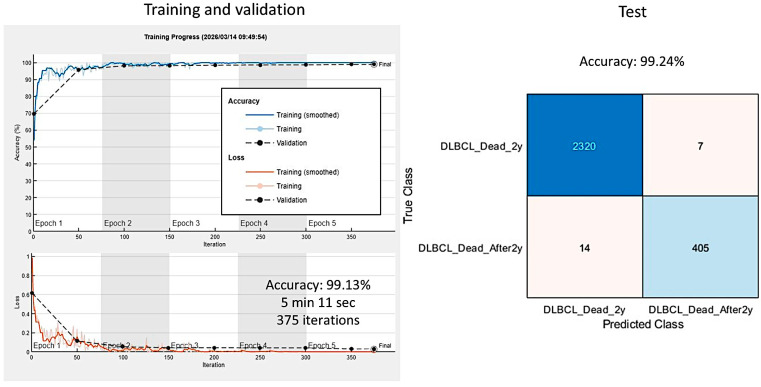
Image classification between Dead < 2 years and Dead > 2 years.

**Figure A2:**
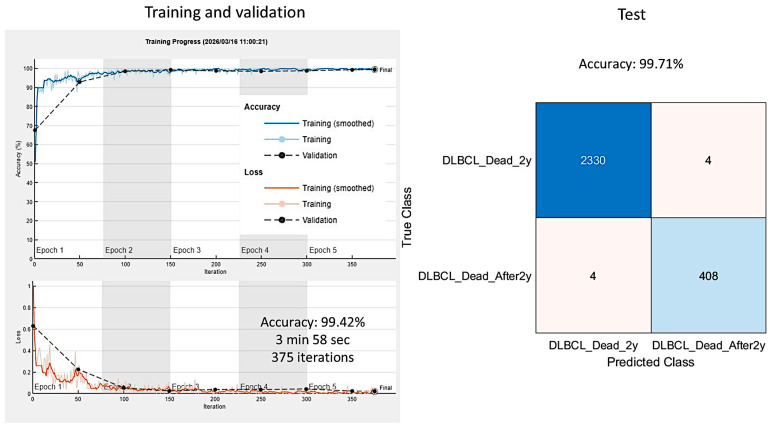
Training/validation and test 1 using training data augmentation of random reflection along the x and y axes.

**Figure A3:**
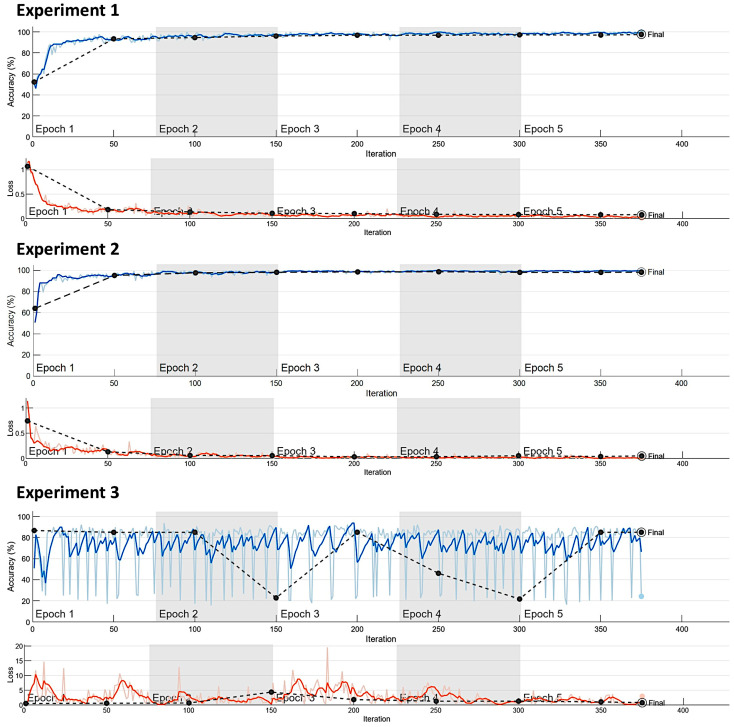
Exhaustive sweep hyperparameter strategy. The hyperparameter learning rate was tuned to train networks under different training conditions and compare the results. For example, sweep through a range of hyperparameter values.

**Figure A4:**
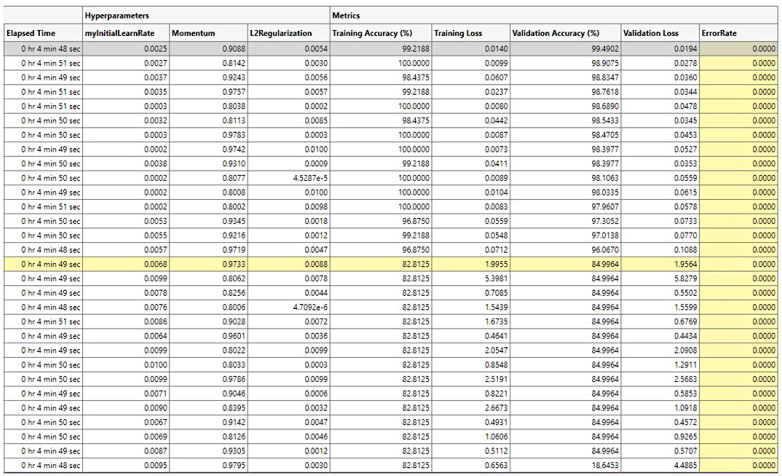
Dead < 2 years vs. >2 years DarkNet19 hyperparameter optimization. The following Bayesian optimization was included: Initial learning rate [1× 10^−4^ 1× 10^−2^], Momentum [0.8 0.98], L2Regularization [1× 10^−10^ 1× 10^−2^], Maximum time (in seconds): Inf, Maximum number of trials: 30, and custom metrics: ErrorRate (direction, minimize).

**Figure A5:**
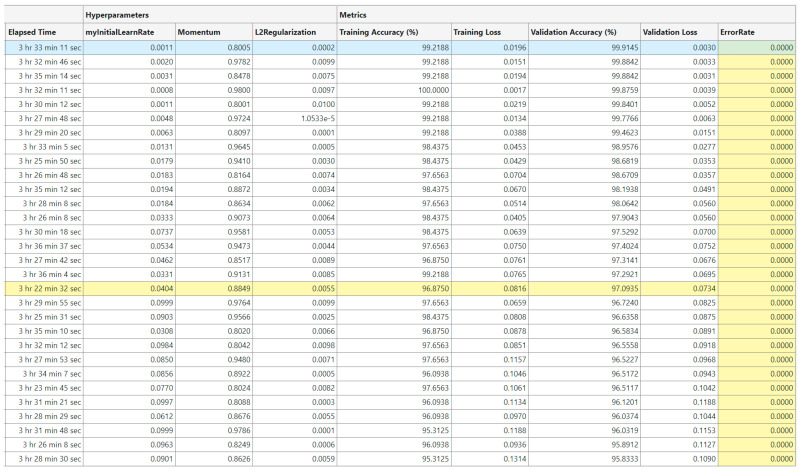
Parameter tuning in DLBCL vs. reactive lymphoid tissue. The analysis of image classification between DLBCL and reactive lymphoid tissue was expanded using DarkNet-19 CNN, data augmentation, and hyperparameter tuning. The following Bayesian optimization was included: Initial learning rate [1× 10^−4^ 1× 10^−2^], Momentum [0.8 0.98], L2Regularization [1× 10^−10^ 1× 10^−2^], Maximum time (in seconds): Inf, Maximum number of trials: 30, and custom metrics: ErrorRate (direction, minimize).

**Figure A6:**
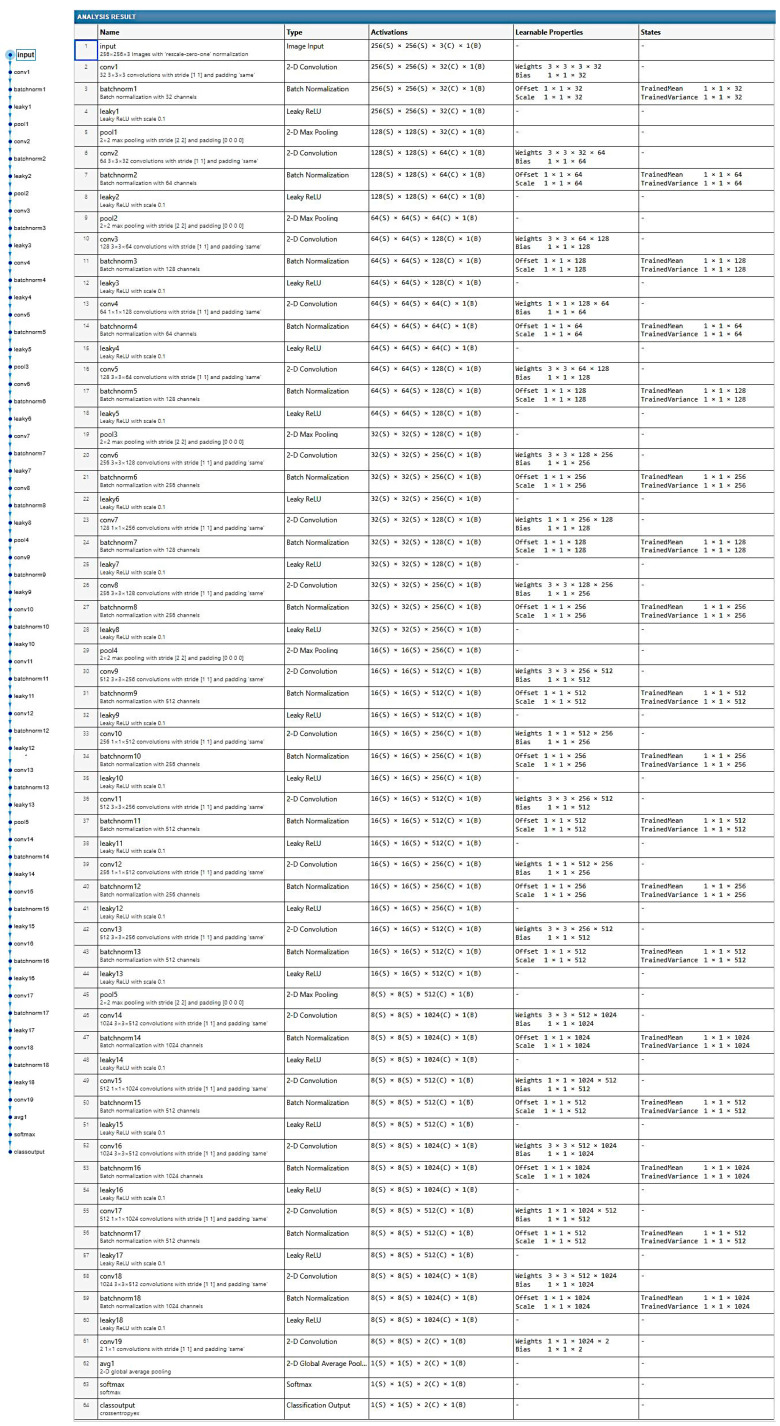
DarkNet-19 architecture. DarkNet-19 is a convolutional neural network (CNN) that is 19 layers deep. This CNN, designed for image classification, is characterized by 19.8 M total learnables, 64 layers and 63 connections.

## Data Availability

All data is available upon request to Joaquim Carreras (joaquim.carreras@tokai.ac.jp) and also shown as Supplementary File.

## Supplementary Materials

The following supporting information can be downloaded at https://www.mdpi.com/article/10.3390/biomedicines14051134/s1.

## Abbreviations

The following abbreviations are used in this manuscript:

CNIO	Spanish National Cancer Research Center
CNN	Convolutional neural network
DLBCL	Diffuse large B-cell lymphoma
H&E	Hematoxylin and eosin
NHL	Non-Hodgkin lymphoma
XAI	Explainable artificial intelligence

## Appendix A

Useful MATLAB R2023b Update 10 (23.2.0.2859533) code:

%L1 is image 1

%% Resize to 299x299x3 because trainedNetwork is Inceptionv3

B = imresize (L1, [299 NaN])

%% Check size of image

sizesz = size(B)

%% Grad-CAM

imds = B

label = classify(trainedNetwork_1,imds)

scoreMap = gradCAM(trainedNetwork_1,imds,label);

figure

imshow(imds)

hold on

imagesc(scoreMap,‘AlphaData’,0.5)

colormap jet

colorbar

%% imageLIME

label = classify(trainedNetwork_1,B)

scoreMap = imageLIME(trainedNetwork_1,B,label);

figure

imshow(B)

hold on

imagesc(scoreMap,‘AlphaData’,0.5)

colormap jet

colorbar

%% OcclusionSensitivity

label = classify(trainedNetwork_1,B)

scoreMap = occlusionSensitivity(trainedNetwork_1,B,label);

figure

imshow(B)

hold on

imagesc(scoreMap,‘AlphaData’,0.5)

colormap jet

colorbar

%% To classify and show image

net = trainedNetwork_1

classes = net.Layers(end).Classes;

[YPred, scores] = classify(net, B);

[score, classIdx] = max(scores);

predClass = classes (classIdx);

imshow(B);

title(sprintf(“%s (%.2f)”,string(predClass),score));

%% To display an image

imshow(B)

%%

imageLoc = “1104802”

imds = imageDatastore(imageLoc)

imds2 = augmentedImageDatastore([299 299 3], imds);

YPred = classify(trainedNetwork_1,imds2)

scores = predict(trainedNetwork_1,imds2)

## Appendix B

Immunohistochemistry was performed using the Leica Bond-Max system and reagents, following the manufacturer’s instruction. Slides were digitized using a NanoZoomer S360 digital slide scanner (Hamamatsu Photonics). Digital image quantification was performed using Fiji software (ImageJ version 1.54f, Wayne Rasband and contributors, National Institutes of Health, USA; Java 1.8.0_322 (64-bit)). The primary antibodies and the staining conditions for the target markers were as follows:

**Table A1:** Immunohistochemical markers and primary antibodies.

Marker	Target	Clone	Company
CD3	T lymphocytes	LN10	Novocastra (Leica)
CD20	B lymphocytes	L26	Novocastra (Leica)
CD5	T lymphocytes	4C7	Novocastra (Leica)
CD10	Germinal center	56C6	Novocastra (Leica)
BCL6	Germinal center	LN22	Novocastra (Leica)
MUM1/IRF4	Plasma cell differentiation	EAU32	Novocastra (Leica)
BCL2	Apoptosis	Bcl2/10/D5	Novocastra (Leica)
EBER	EBV-encoded mRNA	BP0589/AR0833	Novocastra (Leica)
Ki67	Cell proliferation	MM1	Novocastra (Leica)
IL10	Immuno-oncology	LS-B7432	Lifespan Bioscience
PD-L1 (CD274)	Immuno-oncology	E1J2	Cell Signaling
CSF1R	Immuno-oncology	FER216	CNIO
CD163	Tumor-associated macrophages	10D6	Novocastra (Leica)
CASP8	Active subunit p18	11B6	Novocastra
TNFAIP8	Apoptosis	14559-MM0	Sino Biological
LMO2	Hematopoietic development	299B	CNIO
MYC	Proto-oncogene	Y69	Abcam
MDM2	Proto-oncogene	IF2	Invitrogen
CDK6	Cell cycle	98D	CNIO
E2F1	Cell cycle	Agro368V	CNIO
TP53	Cell regulation	DO-7	Novocastra (Leica)

CNIO, Spanish National Cancer Research Center (CNIO), Madrid, Spain.

## Appendix C. Dead < 2 Years vs. Dead > 2 Years

The same type of partition analysis was performed to classify and compare the cases of Dead < 2 years with Dead > 2 years. The analysis included the training, validation, test 1 and test 2 sets, following the hybrid partitioning strategy.

Figure A1 shows the results of the part of the analysis that is made using image patches.

The accuracy of the training/validation was 99.13%, and the test set 1 accuracy was 99.24%. Analysis at the patient level was also performed with 5 cases of Dead < 2 years and 5 cases of Dead > 2 years as test set 2. At the patient level, all 5 cases of Dead < 2 years were correctly classified, but the cases of Dead > 2 years were not (due to the few numbers of cases in the group).

Table A2 shows the clinicopathological characteristics and differences between the two groups. In comparison to Dead > 2 years, Dead < 2 years was characterized by a lower clinical response rate, reduced CD10 and BCL2 protein expressions and a non-GCB phenotype (all *p* values < 0.05).

**Table A2:** Correlation with the clinicopathological characteristics.

Variable	All Dead Cases	Dead < 2 Years (24 months)	Dead > 2 Years (Dead or Alive > 2 Years)	*p* Value
Frequency	54/54 (100%)	38/54 (70.4%)	16/54 (29.5%)	N/A
**Clinical characteristics**				
Age > 60 years	45/54 (83.3%)	30/38 (78.9%)	15/16 (93.8%)	0.253
Male	28/54 (51.9%)	19/38 (50%)	9/16 (56.3%)	0.770
Location				
Nodal (+Spleen)	24/54 (44.4%)	16/38 (42.1%)	8/16 (50%)	0.486
Waldeyer’s ring	4/54 (7.4%)	3/38 (7.9%)	1/16 (6.3%)	
Gastrointestinal	5/54 (48.8%)	5/38 (13.2%)	0/16 (0%)	
Other extranodal	21/54 (38.9%)	14/38 (36.8%)	7/16 (43.8%)	
Stage III–IV		18/28 (64.3%)	6/14 (42.9%)	0.208
IPI High + High/Intermediate	20/41 (48.8%)	14/27 (51.9%)	6/14 (42.9%)	0.744
RCHOP/RCHOP-like treatment		26/28 (92.9%)	13/14 (92.9%)	0.891
Clinical response	39/54 (72.2%)	5/24 (20.8%)	12/14 (85.7%)	<0.001
Hight sIL2R		27/29 (93.1%)	14/15 (93.3%)	1.000
**Pathological characteristics**				
CD3+	0/54 (0%)	0/38 (0%)	0/16 (0%)	1.000
CD20+	54/54 (100%)	38/38 (100%)	16/16 (100%)	1.000
CD5+	7/54 (13.0%)	4/38 (10.5%)	3/16 (18.8%)	0.410
CD10+	7/54 (13.0%)	2/38 (5.3%)	5/16 (31.3%)	0.019
BCL6+	37/54 (68.5%)	26/38 (68.4%)	11/16 (68.8%)	1.000
MUM1+	47/54 (87.0%)	33/38 (86.8%)	14/16 (87.5%)	1.000
Non-GCB	46/54 (85.2%)	35/38 (92.1%)	11/16 (68.8%)	0.041
BCL2+	48/54 (88.9%)	36/38 (94.7%)	12/16 (75.0%)	0.056
*MYC* rearrangement	3/45 (6.7%)	2/29 (6.9%)	1/16 (6.3%)	1.000
EBER+	19/52 (36.5%)	15/37 (40.5%)	4/15 (26.7%)	0.526
Ki67	15.8% ± 12.9	15.7% ± 12.4	16.2% ± 14.5	0.919
Immune microenvironment				
IL10	11.7% ± 14.4	14.7% ± 16.1	5.2% ± 5.9	0.081
PD-L1 (CD274)	13.5% ± 16.1	16.2% ± 18.2	7.8% ± 7.8	0.566
CSF1R	29.7% ± 25.0	27.6% ± 25.3	34.1% ± 24.9	0.405
CD163	39.4 ± 24.9	43.5% ± 23.2	30.6% ± 27.2	0.058
CASP8	5.3% ± 8.1%	6.3% ± 9.5	3.2% ± 3.5	0.738
TNFAIP8	47.2% ± 25.5	46.6% ± 24.5	48.5% ± 28.6	0.685
Cell cycle/GC-related				
LMO2	2.2% ± 3.5	2.3% ± 3.9	1.9% ± 2.2	0.447
MYC	5.3% ± 5.8	6.5% ± 6.4	2.9% ± 3.2	0.080
MDM2	10.0% ± 6.8	9.7% ± 6.1	10.6% ± 8.2	0.964
CDK6	3.8% ± 5.1	3.6% ± 5.3	4.1% ± 4.9	0.178
E2F1	1.3% ± 1.0	1.2% ± 0.9	1.7% ± 1.2	0.210
BCL2	5.5% ± 7.4	3.4% ± 4.5	10.2% ± 10.3	0.022
TP53	5.7% ± 9.0	6.9% ± 10.6	3.2% ± 3.3	0.247

In this series, 8 cases of Alive < 2 years were excluded.

## Appendix D. Parameter Tuning in Dead < 2 Years vs. >2 Years

The analysis of Appendix C was further developed with parameter tuning.

First, data augmentation for the training set was set up using random reflection axes x and y, while leaving the standard training options unchanged, such as the sgdm solver, an initial learning rate of 0.001, a minibatch size of 128, a maximum of 5 epochs, and a validation frequency of 50.

The training and validation lasted for 3 min 58 s, and the validation accuracy was 99.42% at the image patch level. In comparison to the previous analysis shown in Appendix C, the accuracy increased slightly. At the patient level, all 5 cases of Dead < 2 years were correctly classified. However, cases of Dead > 2 years were not; this may be due to the small number of samples in that group. Figure A2 shows the results of the training/validation and test 1 (patch-level analysis). Table A3 and Figure A3 show the exhaustive sweep hyperparameter tuning. Bayesian optimization is shown in Figure A4.

**Table A3:** Exhaustive sweep hyperparameter strategy.

Experiment Details	Exhaustive	Sweep	Hyperparameters	Metrics			
Experiment 1	Status	Elapsed Time	Initial Learning Rate	Training Accuracy	Training Loss	Validation Accuracy	Validation Loss
1	Max epochs completed	4 min 50 s	0.0001	100.0000	0.0151	97.6693	0.0735
2	Max epochs completed	4 min 48 s	0.0010	99.2188	0.0159	98.5433	0.0478
3	Max epochs completed	4 min 48 s	0.0100	24.2188	3.0129	84.9964	0.8352

The hyperparameter learning rate was tuned to train networks under different training conditions and compare the results. For example, sweep through a range of hyperparameter values.

## Appendix E. Parameter Tuning in DLBCL vs. Reactive

The analysis of image classification between DLBCL and reactive lymphoid tissue was expanded using DarkNet-19 CNN, data augmentation, and hyperparameter tuning. The following Bayesian optimization was included: Initial learning rate [1× 10^−4^ 1× 10^−2^], Momentum [0.8 0.98], L2Regularization [1× 10^−10^ 1× 10^−2^], Maximum time (in seconds): Inf, Maximum number of trials: 30, and custom metrics: ErrorRate (direction, minimize) (Figure A5).

## Appendix F. DarkNet-19 Architecture

The analysis was performed using several CNNs, and DarkNet-19 was the CNN with best performance taking into account the accuracy and training time. The architecture of DarkNet-19 is shown in Figure A6.

## Appendix G. CNN with Data Augmentation

A small experiment was conducted with the ResNet18 CNN because it had a relative time of 1.4 (with respect to AlexNet, which was the fastest) but still had a high validation accuracy of 92.11% during training. The CNN image classification experiment was run with and without augmentation (Figure A7). The augmentation steps included random refection axis (x and y), random rotation (degrees)—min 0 to max 30, random rescaling (none)—min 1 to max 1, random horizontal translation (pixels)—min 0 to max 0.2, and random vertical translation (pixels)—min 0 to max 0.2. The rest of the parameters were the same as described in the paper. The results showed that with augmentation, the validation accuracy during training was 90.3% and the test accuracy was 90.39%. Without augmentation the validation accuracy was 92.02% and the test accuracy was 92.09%. Therefore, the results augmentation showed a slightly lower accuracy.

## Appendix H. Training Options and Hyperparameters

**Table A4:** Training options and hyperparameters.

Training Options and Hyperparameters
Input type: image patches Output type: classification Solver: sgdm Initial learning rate: 0.001 MiniBatch size: 128 MaxEpochs: 5 Validation frequency: 50 Additional training parameters. Additional detailed training options Import images Augmentation options: none Available parameters Random reflection axis: x, y Random rotation (degrees): min, max Random rescaling: min, max Random horizontal translation (pixels): min, max Random vertical translation (pixels): min, max Resize during training to match network input size: yes, no Solver Momentum: 0.9 Learn rate LearnRateSchedule: none LearnRateDropFactor: 0.1 LearnRateDropPeriod: 10 Normalization and Regularization L2Regularization: 0.0001 ResetInputNormalization: yes BatchNormalizationStatistics: population Mini-Batch Shuffle: every-epoch Validation and Output ValidationPatience: Inf OutputNetwork: last-iteration Gradient Clipping GradientThresholdMethod: I2norm GradientThreshold: Inf Hardware ExecutionThreshold: auto. Checkpoint CheckpointPath: n/a CheckpointFrequency: 1 CheckpointFrequencyUnit: epoch
