# Supplementary figures and images for: Clinicopathological Characteristics and Prediction of Overall Survival and Death Within 2 Years in Diffuse Large B-Cell Lymphoma Based on Histological Images and Deep Learning (part 2 of 2)
Source: Biomedicines. 2026 May 17;14(5):1134. doi: 10.3390/biomedicines14051134 (PMC13205024; doi:10.3390/biomedicines14051134)

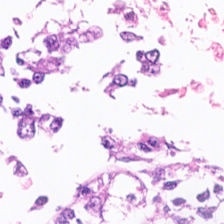

Supplement: Supplementary file 1 [file biomedicines-14-01134-s001.zip › Images_examples_v20260307_1507/Others/Others (46).jpg]

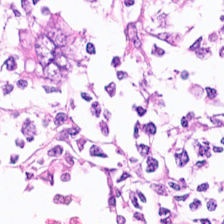

Supplement: Supplementary file 1 [file biomedicines-14-01134-s001.zip › Images_examples_v20260307_1507/Others/Others (47).jpg]

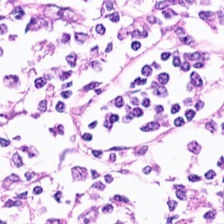

Supplement: Supplementary file 1 [file biomedicines-14-01134-s001.zip › Images_examples_v20260307_1507/Others/Others (48).jpg]

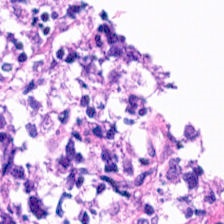

Supplement: Supplementary file 1 [file biomedicines-14-01134-s001.zip › Images_examples_v20260307_1507/Others/Others (5).jpg]

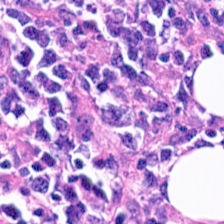

Supplement: Supplementary file 1 [file biomedicines-14-01134-s001.zip › Images_examples_v20260307_1507/Others/Others (6).jpg]

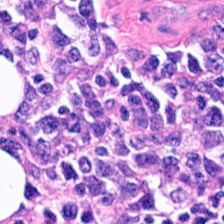

Supplement: Supplementary file 1 [file biomedicines-14-01134-s001.zip › Images_examples_v20260307_1507/Others/Others (7).jpg]

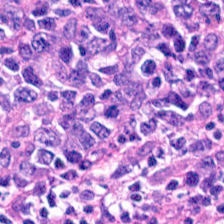

Supplement: Supplementary file 1 [file biomedicines-14-01134-s001.zip › Images_examples_v20260307_1507/Others/Others (8).jpg]

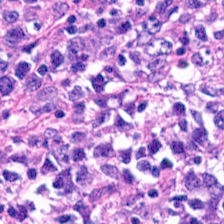

Supplement: Supplementary file 1 [file biomedicines-14-01134-s001.zip › Images_examples_v20260307_1507/Others/Others (9).jpg]

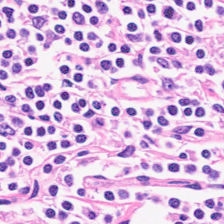

Supplement: Supplementary file 1 [file biomedicines-14-01134-s001.zip › Images_examples_v20260307_1507/Reactive/Reactive (1).jpg]

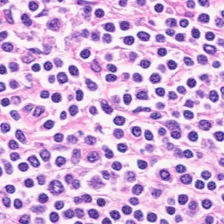

Supplement: Supplementary file 1 [file biomedicines-14-01134-s001.zip › Images_examples_v20260307_1507/Reactive/Reactive (10).jpg]

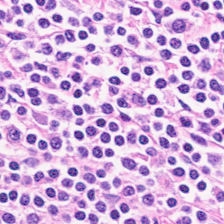

Supplement: Supplementary file 1 [file biomedicines-14-01134-s001.zip › Images_examples_v20260307_1507/Reactive/Reactive (11).jpg]

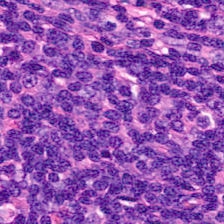

Supplement: Supplementary file 1 [file biomedicines-14-01134-s001.zip › Images_examples_v20260307_1507/Reactive/Reactive (12).jpg]

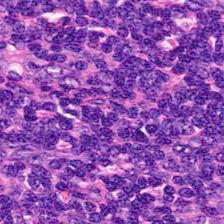

Supplement: Supplementary file 1 [file biomedicines-14-01134-s001.zip › Images_examples_v20260307_1507/Reactive/Reactive (13).jpg]

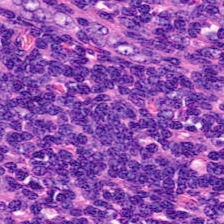

Supplement: Supplementary file 1 [file biomedicines-14-01134-s001.zip › Images_examples_v20260307_1507/Reactive/Reactive (14).jpg]

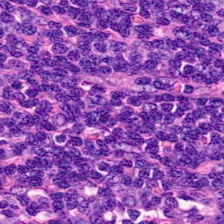

Supplement: Supplementary file 1 [file biomedicines-14-01134-s001.zip › Images_examples_v20260307_1507/Reactive/Reactive (15).jpg]

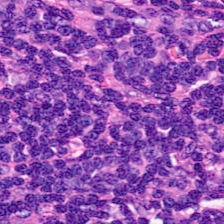

Supplement: Supplementary file 1 [file biomedicines-14-01134-s001.zip › Images_examples_v20260307_1507/Reactive/Reactive (16).jpg]

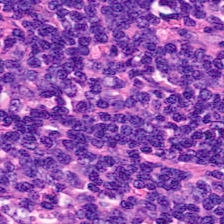

Supplement: Supplementary file 1 [file biomedicines-14-01134-s001.zip › Images_examples_v20260307_1507/Reactive/Reactive (17).jpg]

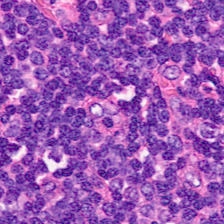

Supplement: Supplementary file 1 [file biomedicines-14-01134-s001.zip › Images_examples_v20260307_1507/Reactive/Reactive (18).jpg]

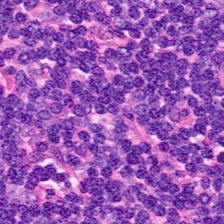

Supplement: Supplementary file 1 [file biomedicines-14-01134-s001.zip › Images_examples_v20260307_1507/Reactive/Reactive (19).jpg]

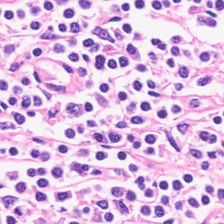

Supplement: Supplementary file 1 [file biomedicines-14-01134-s001.zip › Images_examples_v20260307_1507/Reactive/Reactive (2).jpg]

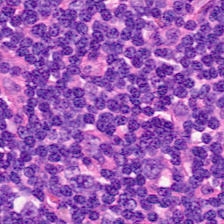

Supplement: Supplementary file 1 [file biomedicines-14-01134-s001.zip › Images_examples_v20260307_1507/Reactive/Reactive (20).jpg]

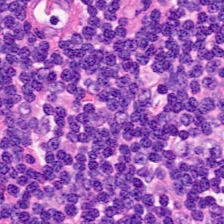

Supplement: Supplementary file 1 [file biomedicines-14-01134-s001.zip › Images_examples_v20260307_1507/Reactive/Reactive (21).jpg]

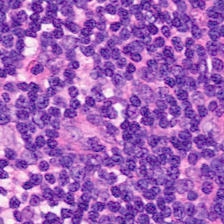

Supplement: Supplementary file 1 [file biomedicines-14-01134-s001.zip › Images_examples_v20260307_1507/Reactive/Reactive (22).jpg]

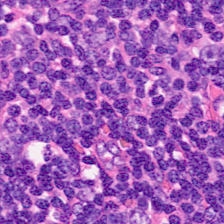

Supplement: Supplementary file 1 [file biomedicines-14-01134-s001.zip › Images_examples_v20260307_1507/Reactive/Reactive (23).jpg]

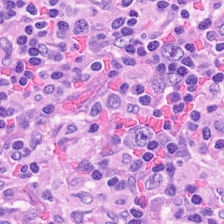

Supplement: Supplementary file 1 [file biomedicines-14-01134-s001.zip › Images_examples_v20260307_1507/Reactive/Reactive (24).jpg]

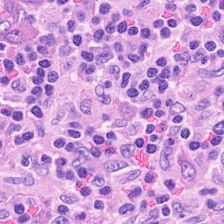

Supplement: Supplementary file 1 [file biomedicines-14-01134-s001.zip › Images_examples_v20260307_1507/Reactive/Reactive (25).jpg]

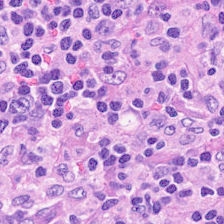

Supplement: Supplementary file 1 [file biomedicines-14-01134-s001.zip › Images_examples_v20260307_1507/Reactive/Reactive (26).jpg]

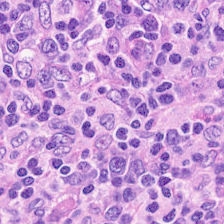

Supplement: Supplementary file 1 [file biomedicines-14-01134-s001.zip › Images_examples_v20260307_1507/Reactive/Reactive (27).jpg]

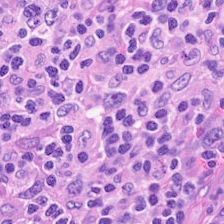

Supplement: Supplementary file 1 [file biomedicines-14-01134-s001.zip › Images_examples_v20260307_1507/Reactive/Reactive (28).jpg]

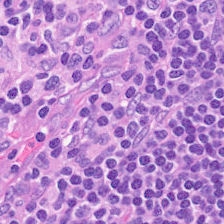

Supplement: Supplementary file 1 [file biomedicines-14-01134-s001.zip › Images_examples_v20260307_1507/Reactive/Reactive (29).jpg]

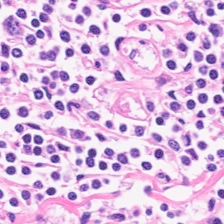

Supplement: Supplementary file 1 [file biomedicines-14-01134-s001.zip › Images_examples_v20260307_1507/Reactive/Reactive (3).jpg]

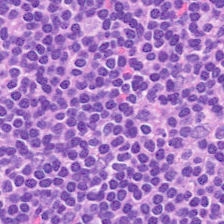

Supplement: Supplementary file 1 [file biomedicines-14-01134-s001.zip › Images_examples_v20260307_1507/Reactive/Reactive (30).jpg]

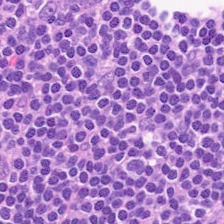

Supplement: Supplementary file 1 [file biomedicines-14-01134-s001.zip › Images_examples_v20260307_1507/Reactive/Reactive (31).jpg]

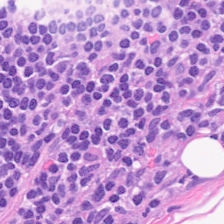

Supplement: Supplementary file 1 [file biomedicines-14-01134-s001.zip › Images_examples_v20260307_1507/Reactive/Reactive (32).jpg]

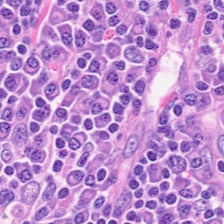

Supplement: Supplementary file 1 [file biomedicines-14-01134-s001.zip › Images_examples_v20260307_1507/Reactive/Reactive (33).jpg]

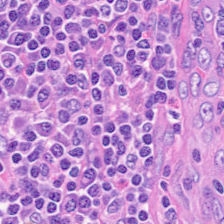

Supplement: Supplementary file 1 [file biomedicines-14-01134-s001.zip › Images_examples_v20260307_1507/Reactive/Reactive (34).jpg]

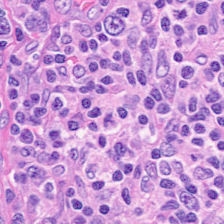

Supplement: Supplementary file 1 [file biomedicines-14-01134-s001.zip › Images_examples_v20260307_1507/Reactive/Reactive (35).jpg]

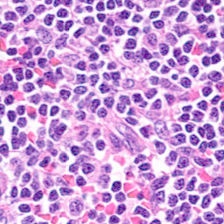

Supplement: Supplementary file 1 [file biomedicines-14-01134-s001.zip › Images_examples_v20260307_1507/Reactive/Reactive (36).jpg]

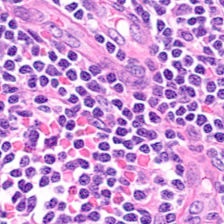

Supplement: Supplementary file 1 [file biomedicines-14-01134-s001.zip › Images_examples_v20260307_1507/Reactive/Reactive (37).jpg]

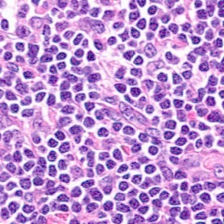

Supplement: Supplementary file 1 [file biomedicines-14-01134-s001.zip › Images_examples_v20260307_1507/Reactive/Reactive (38).jpg]

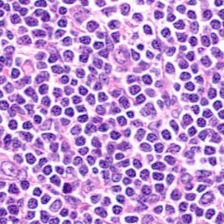

Supplement: Supplementary file 1 [file biomedicines-14-01134-s001.zip › Images_examples_v20260307_1507/Reactive/Reactive (39).jpg]

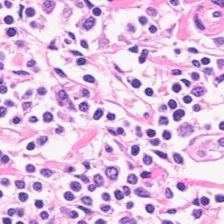

Supplement: Supplementary file 1 [file biomedicines-14-01134-s001.zip › Images_examples_v20260307_1507/Reactive/Reactive (4).jpg]

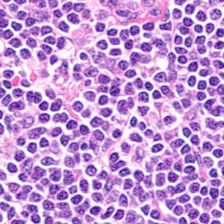

Supplement: Supplementary file 1 [file biomedicines-14-01134-s001.zip › Images_examples_v20260307_1507/Reactive/Reactive (40).jpg]

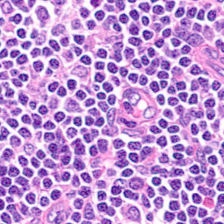

Supplement: Supplementary file 1 [file biomedicines-14-01134-s001.zip › Images_examples_v20260307_1507/Reactive/Reactive (41).jpg]

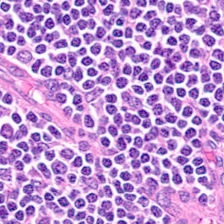

Supplement: Supplementary file 1 [file biomedicines-14-01134-s001.zip › Images_examples_v20260307_1507/Reactive/Reactive (42).jpg]

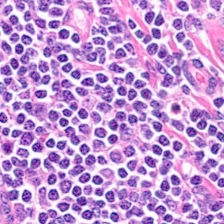

Supplement: Supplementary file 1 [file biomedicines-14-01134-s001.zip › Images_examples_v20260307_1507/Reactive/Reactive (43).jpg]

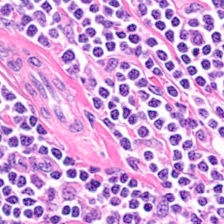

Supplement: Supplementary file 1 [file biomedicines-14-01134-s001.zip › Images_examples_v20260307_1507/Reactive/Reactive (44).jpg]

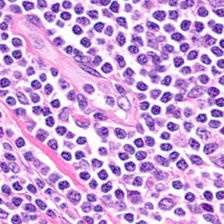

Supplement: Supplementary file 1 [file biomedicines-14-01134-s001.zip › Images_examples_v20260307_1507/Reactive/Reactive (45).jpg]

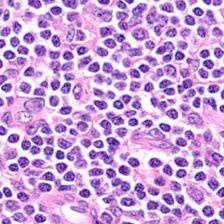

Supplement: Supplementary file 1 [file biomedicines-14-01134-s001.zip › Images_examples_v20260307_1507/Reactive/Reactive (46).jpg]

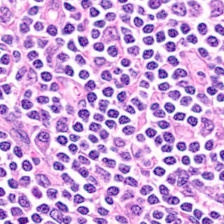

Supplement: Supplementary file 1 [file biomedicines-14-01134-s001.zip › Images_examples_v20260307_1507/Reactive/Reactive (47).jpg]

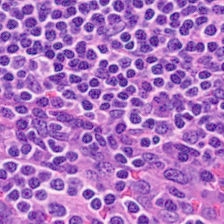

Supplement: Supplementary file 1 [file biomedicines-14-01134-s001.zip › Images_examples_v20260307_1507/Reactive/Reactive (48).jpg]

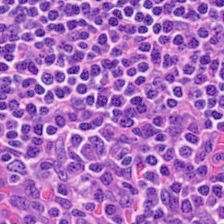

Supplement: Supplementary file 1 [file biomedicines-14-01134-s001.zip › Images_examples_v20260307_1507/Reactive/Reactive (49).jpg]

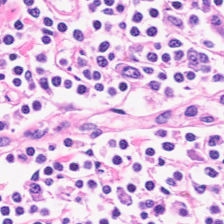

Supplement: Supplementary file 1 [file biomedicines-14-01134-s001.zip › Images_examples_v20260307_1507/Reactive/Reactive (5).jpg]

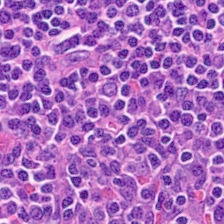

Supplement: Supplementary file 1 [file biomedicines-14-01134-s001.zip › Images_examples_v20260307_1507/Reactive/Reactive (50).jpg]

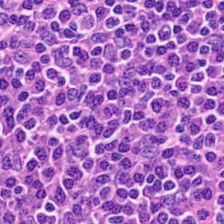

Supplement: Supplementary file 1 [file biomedicines-14-01134-s001.zip › Images_examples_v20260307_1507/Reactive/Reactive (51).jpg]

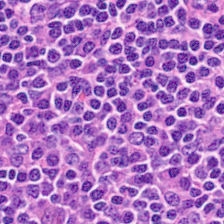

Supplement: Supplementary file 1 [file biomedicines-14-01134-s001.zip › Images_examples_v20260307_1507/Reactive/Reactive (52).jpg]

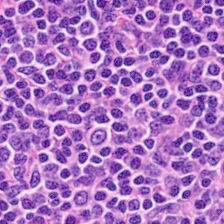

Supplement: Supplementary file 1 [file biomedicines-14-01134-s001.zip › Images_examples_v20260307_1507/Reactive/Reactive (53).jpg]

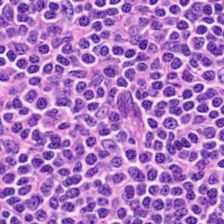

Supplement: Supplementary file 1 [file biomedicines-14-01134-s001.zip › Images_examples_v20260307_1507/Reactive/Reactive (54).jpg]

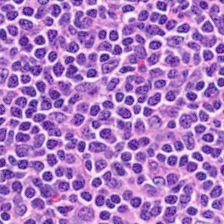

Supplement: Supplementary file 1 [file biomedicines-14-01134-s001.zip › Images_examples_v20260307_1507/Reactive/Reactive (55).jpg]

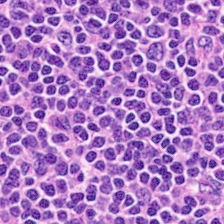

Supplement: Supplementary file 1 [file biomedicines-14-01134-s001.zip › Images_examples_v20260307_1507/Reactive/Reactive (56).jpg]

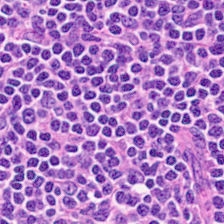

Supplement: Supplementary file 1 [file biomedicines-14-01134-s001.zip › Images_examples_v20260307_1507/Reactive/Reactive (57).jpg]

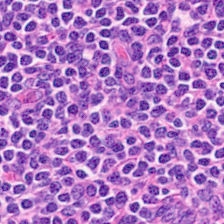

Supplement: Supplementary file 1 [file biomedicines-14-01134-s001.zip › Images_examples_v20260307_1507/Reactive/Reactive (58).jpg]

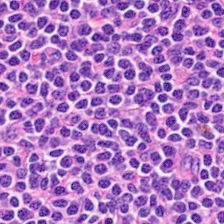

Supplement: Supplementary file 1 [file biomedicines-14-01134-s001.zip › Images_examples_v20260307_1507/Reactive/Reactive (59).jpg]

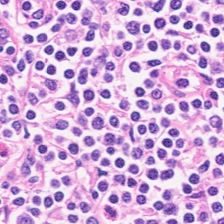

Supplement: Supplementary file 1 [file biomedicines-14-01134-s001.zip › Images_examples_v20260307_1507/Reactive/Reactive (6).jpg]

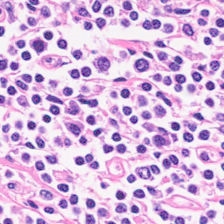

Supplement: Supplementary file 1 [file biomedicines-14-01134-s001.zip › Images_examples_v20260307_1507/Reactive/Reactive (60).jpg]

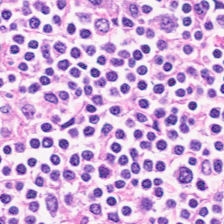

Supplement: Supplementary file 1 [file biomedicines-14-01134-s001.zip › Images_examples_v20260307_1507/Reactive/Reactive (7).jpg]

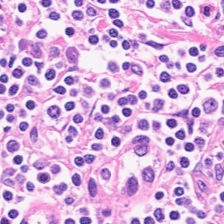

Supplement: Supplementary file 1 [file biomedicines-14-01134-s001.zip › Images_examples_v20260307_1507/Reactive/Reactive (8).jpg]

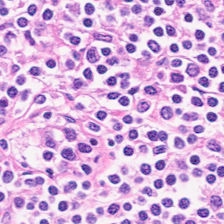

Supplement: Supplementary file 1 [file biomedicines-14-01134-s001.zip › Images_examples_v20260307_1507/Reactive/Reactive (9).jpg]
