# Supplementary figures and images for: Clinicopathological Characteristics and Prediction of Overall Survival and Death Within 2 Years in Diffuse Large B-Cell Lymphoma Based on Histological Images and Deep Learning (part 1 of 2)
Source: Biomedicines. 2026 May 17;14(5):1134. doi: 10.3390/biomedicines14051134 (PMC13205024; doi:10.3390/biomedicines14051134)

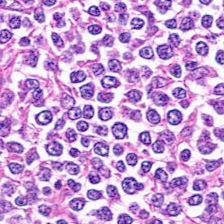

Supplement: Supplementary file 1 [file biomedicines-14-01134-s001.zip › Images_examples_v20260307_1507/D2Y/D2Y (1).jpg]

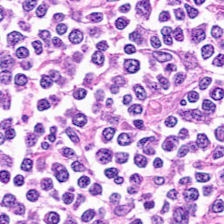

Supplement: Supplementary file 1 [file biomedicines-14-01134-s001.zip › Images_examples_v20260307_1507/D2Y/D2Y (10).jpg]

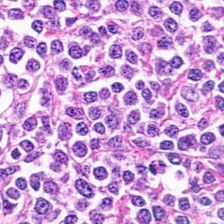

Supplement: Supplementary file 1 [file biomedicines-14-01134-s001.zip › Images_examples_v20260307_1507/D2Y/D2Y (11).jpg]

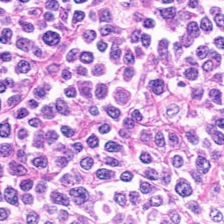

Supplement: Supplementary file 1 [file biomedicines-14-01134-s001.zip › Images_examples_v20260307_1507/D2Y/D2Y (12).jpg]

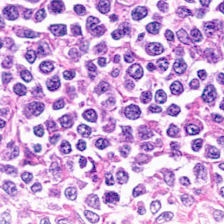

Supplement: Supplementary file 1 [file biomedicines-14-01134-s001.zip › Images_examples_v20260307_1507/D2Y/D2Y (13).jpg]

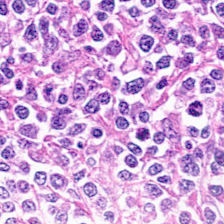

Supplement: Supplementary file 1 [file biomedicines-14-01134-s001.zip › Images_examples_v20260307_1507/D2Y/D2Y (14).jpg]

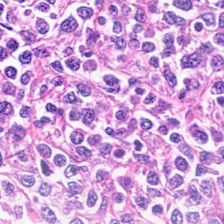

Supplement: Supplementary file 1 [file biomedicines-14-01134-s001.zip › Images_examples_v20260307_1507/D2Y/D2Y (15).jpg]

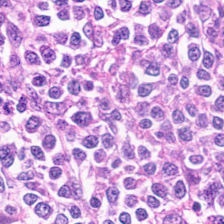

Supplement: Supplementary file 1 [file biomedicines-14-01134-s001.zip › Images_examples_v20260307_1507/D2Y/D2Y (16).jpg]

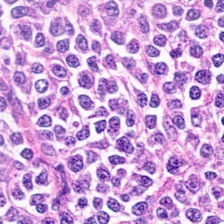

Supplement: Supplementary file 1 [file biomedicines-14-01134-s001.zip › Images_examples_v20260307_1507/D2Y/D2Y (17).jpg]

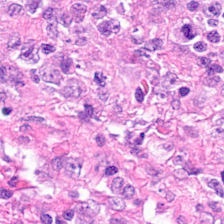

Supplement: Supplementary file 1 [file biomedicines-14-01134-s001.zip › Images_examples_v20260307_1507/D2Y/D2Y (18).jpg]

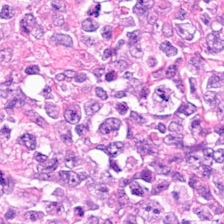

Supplement: Supplementary file 1 [file biomedicines-14-01134-s001.zip › Images_examples_v20260307_1507/D2Y/D2Y (19).jpg]

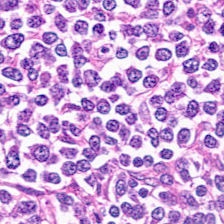

Supplement: Supplementary file 1 [file biomedicines-14-01134-s001.zip › Images_examples_v20260307_1507/D2Y/D2Y (2).jpg]

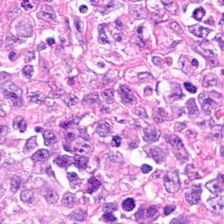

Supplement: Supplementary file 1 [file biomedicines-14-01134-s001.zip › Images_examples_v20260307_1507/D2Y/D2Y (20).jpg]

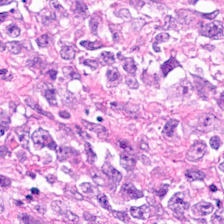

Supplement: Supplementary file 1 [file biomedicines-14-01134-s001.zip › Images_examples_v20260307_1507/D2Y/D2Y (21).jpg]

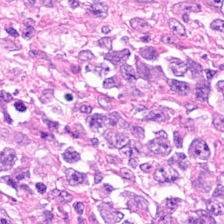

Supplement: Supplementary file 1 [file biomedicines-14-01134-s001.zip › Images_examples_v20260307_1507/D2Y/D2Y (22).jpg]

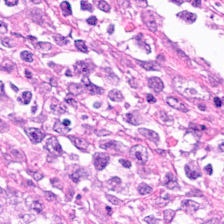

Supplement: Supplementary file 1 [file biomedicines-14-01134-s001.zip › Images_examples_v20260307_1507/D2Y/D2Y (23).jpg]

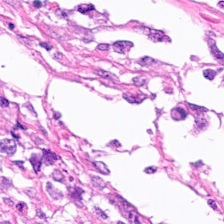

Supplement: Supplementary file 1 [file biomedicines-14-01134-s001.zip › Images_examples_v20260307_1507/D2Y/D2Y (24).jpg]

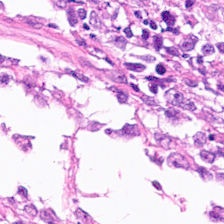

Supplement: Supplementary file 1 [file biomedicines-14-01134-s001.zip › Images_examples_v20260307_1507/D2Y/D2Y (25).jpg]

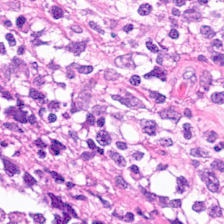

Supplement: Supplementary file 1 [file biomedicines-14-01134-s001.zip › Images_examples_v20260307_1507/D2Y/D2Y (26).jpg]

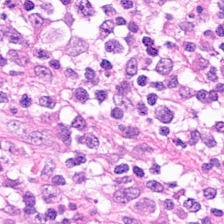

Supplement: Supplementary file 1 [file biomedicines-14-01134-s001.zip › Images_examples_v20260307_1507/D2Y/D2Y (27).jpg]

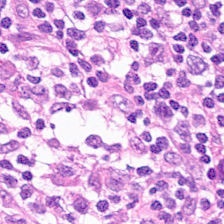

Supplement: Supplementary file 1 [file biomedicines-14-01134-s001.zip › Images_examples_v20260307_1507/D2Y/D2Y (28).jpg]

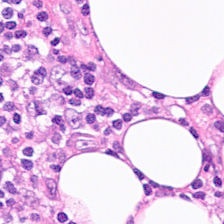

Supplement: Supplementary file 1 [file biomedicines-14-01134-s001.zip › Images_examples_v20260307_1507/D2Y/D2Y (29).jpg]

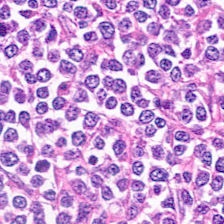

Supplement: Supplementary file 1 [file biomedicines-14-01134-s001.zip › Images_examples_v20260307_1507/D2Y/D2Y (3).jpg]

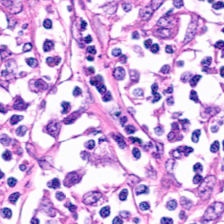

Supplement: Supplementary file 1 [file biomedicines-14-01134-s001.zip › Images_examples_v20260307_1507/D2Y/D2Y (30).jpg]

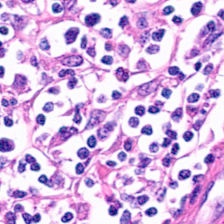

Supplement: Supplementary file 1 [file biomedicines-14-01134-s001.zip › Images_examples_v20260307_1507/D2Y/D2Y (31).jpg]

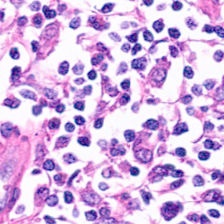

Supplement: Supplementary file 1 [file biomedicines-14-01134-s001.zip › Images_examples_v20260307_1507/D2Y/D2Y (32).jpg]

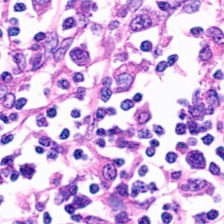

Supplement: Supplementary file 1 [file biomedicines-14-01134-s001.zip › Images_examples_v20260307_1507/D2Y/D2Y (33).jpg]

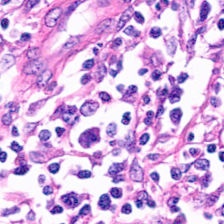

Supplement: Supplementary file 1 [file biomedicines-14-01134-s001.zip › Images_examples_v20260307_1507/D2Y/D2Y (34).jpg]

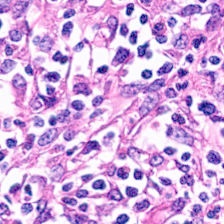

Supplement: Supplementary file 1 [file biomedicines-14-01134-s001.zip › Images_examples_v20260307_1507/D2Y/D2Y (35).jpg]

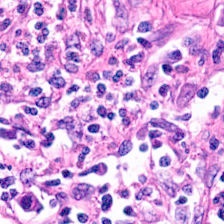

Supplement: Supplementary file 1 [file biomedicines-14-01134-s001.zip › Images_examples_v20260307_1507/D2Y/D2Y (36).jpg]

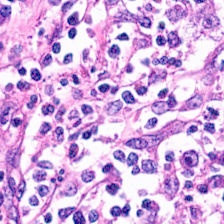

Supplement: Supplementary file 1 [file biomedicines-14-01134-s001.zip › Images_examples_v20260307_1507/D2Y/D2Y (37).jpg]

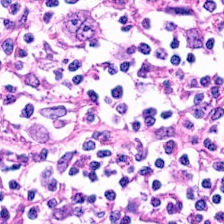

Supplement: Supplementary file 1 [file biomedicines-14-01134-s001.zip › Images_examples_v20260307_1507/D2Y/D2Y (38).jpg]

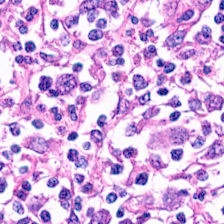

Supplement: Supplementary file 1 [file biomedicines-14-01134-s001.zip › Images_examples_v20260307_1507/D2Y/D2Y (39).jpg]

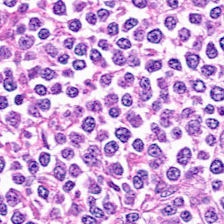

Supplement: Supplementary file 1 [file biomedicines-14-01134-s001.zip › Images_examples_v20260307_1507/D2Y/D2Y (4).jpg]

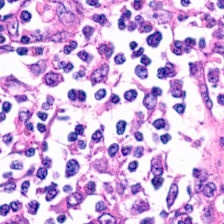

Supplement: Supplementary file 1 [file biomedicines-14-01134-s001.zip › Images_examples_v20260307_1507/D2Y/D2Y (40).jpg]

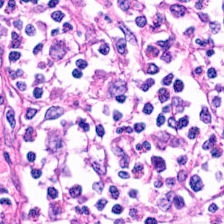

Supplement: Supplementary file 1 [file biomedicines-14-01134-s001.zip › Images_examples_v20260307_1507/D2Y/D2Y (41).jpg]

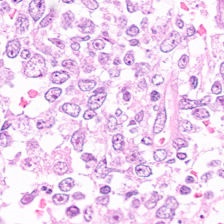

Supplement: Supplementary file 1 [file biomedicines-14-01134-s001.zip › Images_examples_v20260307_1507/D2Y/D2Y (42).jpg]

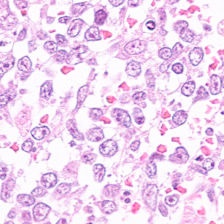

Supplement: Supplementary file 1 [file biomedicines-14-01134-s001.zip › Images_examples_v20260307_1507/D2Y/D2Y (43).jpg]

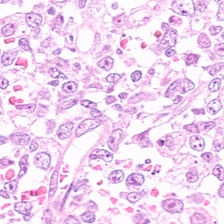

Supplement: Supplementary file 1 [file biomedicines-14-01134-s001.zip › Images_examples_v20260307_1507/D2Y/D2Y (44).jpg]

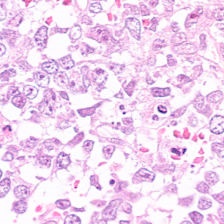

Supplement: Supplementary file 1 [file biomedicines-14-01134-s001.zip › Images_examples_v20260307_1507/D2Y/D2Y (45).jpg]

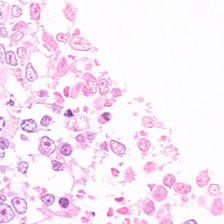

Supplement: Supplementary file 1 [file biomedicines-14-01134-s001.zip › Images_examples_v20260307_1507/D2Y/D2Y (46).jpg]

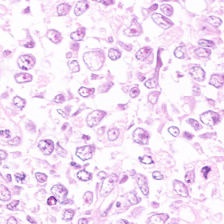

Supplement: Supplementary file 1 [file biomedicines-14-01134-s001.zip › Images_examples_v20260307_1507/D2Y/D2Y (47).jpg]

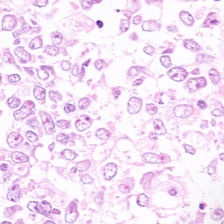

Supplement: Supplementary file 1 [file biomedicines-14-01134-s001.zip › Images_examples_v20260307_1507/D2Y/D2Y (48).jpg]

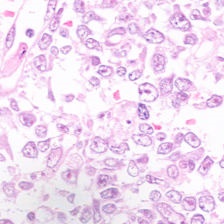

Supplement: Supplementary file 1 [file biomedicines-14-01134-s001.zip › Images_examples_v20260307_1507/D2Y/D2Y (49).jpg]

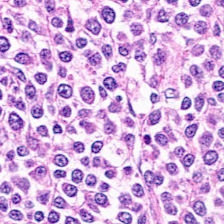

Supplement: Supplementary file 1 [file biomedicines-14-01134-s001.zip › Images_examples_v20260307_1507/D2Y/D2Y (5).jpg]

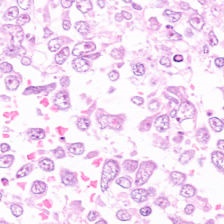

Supplement: Supplementary file 1 [file biomedicines-14-01134-s001.zip › Images_examples_v20260307_1507/D2Y/D2Y (50).jpg]

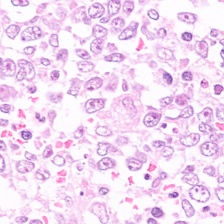

Supplement: Supplementary file 1 [file biomedicines-14-01134-s001.zip › Images_examples_v20260307_1507/D2Y/D2Y (51).jpg]

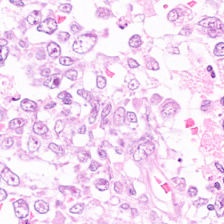

Supplement: Supplementary file 1 [file biomedicines-14-01134-s001.zip › Images_examples_v20260307_1507/D2Y/D2Y (52).jpg]

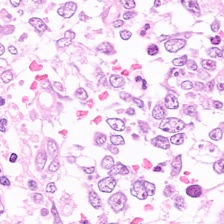

Supplement: Supplementary file 1 [file biomedicines-14-01134-s001.zip › Images_examples_v20260307_1507/D2Y/D2Y (53).jpg]

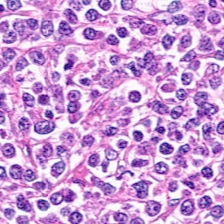

Supplement: Supplementary file 1 [file biomedicines-14-01134-s001.zip › Images_examples_v20260307_1507/D2Y/D2Y (54).jpg]

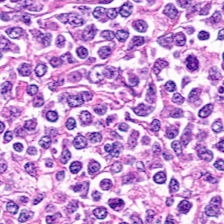

Supplement: Supplementary file 1 [file biomedicines-14-01134-s001.zip › Images_examples_v20260307_1507/D2Y/D2Y (55).jpg]

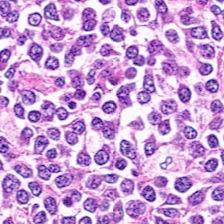

Supplement: Supplementary file 1 [file biomedicines-14-01134-s001.zip › Images_examples_v20260307_1507/D2Y/D2Y (56).jpg]

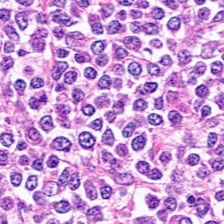

Supplement: Supplementary file 1 [file biomedicines-14-01134-s001.zip › Images_examples_v20260307_1507/D2Y/D2Y (57).jpg]

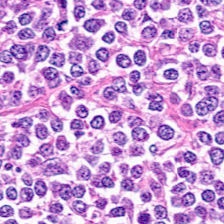

Supplement: Supplementary file 1 [file biomedicines-14-01134-s001.zip › Images_examples_v20260307_1507/D2Y/D2Y (58).jpg]

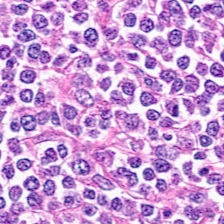

Supplement: Supplementary file 1 [file biomedicines-14-01134-s001.zip › Images_examples_v20260307_1507/D2Y/D2Y (59).jpg]

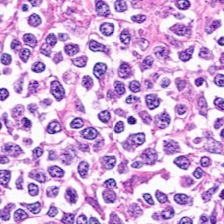

Supplement: Supplementary file 1 [file biomedicines-14-01134-s001.zip › Images_examples_v20260307_1507/D2Y/D2Y (6).jpg]

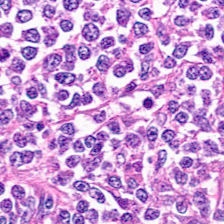

Supplement: Supplementary file 1 [file biomedicines-14-01134-s001.zip › Images_examples_v20260307_1507/D2Y/D2Y (60).jpg]

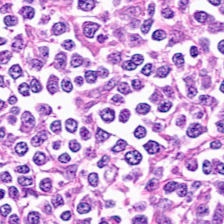

Supplement: Supplementary file 1 [file biomedicines-14-01134-s001.zip › Images_examples_v20260307_1507/D2Y/D2Y (7).jpg]

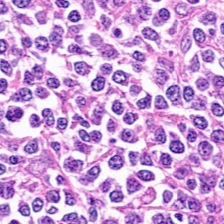

Supplement: Supplementary file 1 [file biomedicines-14-01134-s001.zip › Images_examples_v20260307_1507/D2Y/D2Y (8).jpg]

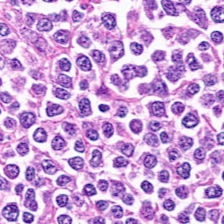

Supplement: Supplementary file 1 [file biomedicines-14-01134-s001.zip › Images_examples_v20260307_1507/D2Y/D2Y (9).jpg]

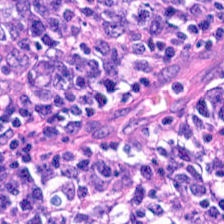

Supplement: Supplementary file 1 [file biomedicines-14-01134-s001.zip › Images_examples_v20260307_1507/Others/Others (1).jpg]

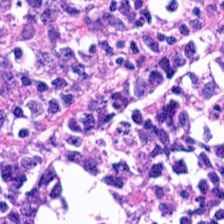

Supplement: Supplementary file 1 [file biomedicines-14-01134-s001.zip › Images_examples_v20260307_1507/Others/Others (10).jpg]

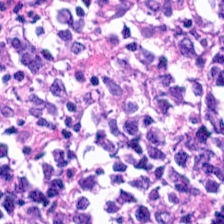

Supplement: Supplementary file 1 [file biomedicines-14-01134-s001.zip › Images_examples_v20260307_1507/Others/Others (11).jpg]

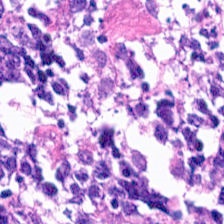

Supplement: Supplementary file 1 [file biomedicines-14-01134-s001.zip › Images_examples_v20260307_1507/Others/Others (12).jpg]

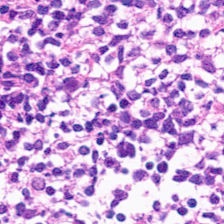

Supplement: Supplementary file 1 [file biomedicines-14-01134-s001.zip › Images_examples_v20260307_1507/Others/Others (13).jpg]

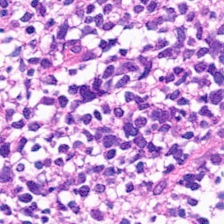

Supplement: Supplementary file 1 [file biomedicines-14-01134-s001.zip › Images_examples_v20260307_1507/Others/Others (14).jpg]

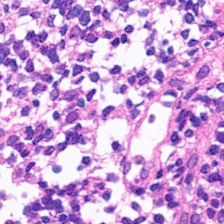

Supplement: Supplementary file 1 [file biomedicines-14-01134-s001.zip › Images_examples_v20260307_1507/Others/Others (15).jpg]

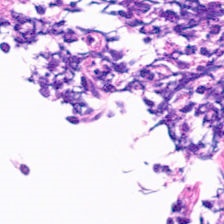

Supplement: Supplementary file 1 [file biomedicines-14-01134-s001.zip › Images_examples_v20260307_1507/Others/Others (16).jpg]

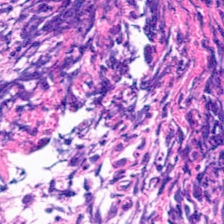

Supplement: Supplementary file 1 [file biomedicines-14-01134-s001.zip › Images_examples_v20260307_1507/Others/Others (17).jpg]

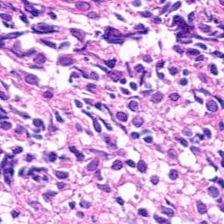

Supplement: Supplementary file 1 [file biomedicines-14-01134-s001.zip › Images_examples_v20260307_1507/Others/Others (18).jpg]

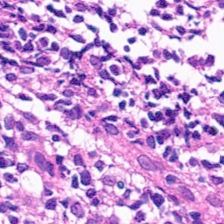

Supplement: Supplementary file 1 [file biomedicines-14-01134-s001.zip › Images_examples_v20260307_1507/Others/Others (19).jpg]

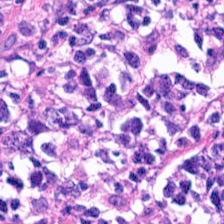

Supplement: Supplementary file 1 [file biomedicines-14-01134-s001.zip › Images_examples_v20260307_1507/Others/Others (2).jpg]

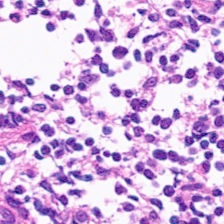

Supplement: Supplementary file 1 [file biomedicines-14-01134-s001.zip › Images_examples_v20260307_1507/Others/Others (20).jpg]

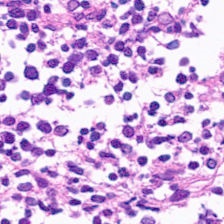

Supplement: Supplementary file 1 [file biomedicines-14-01134-s001.zip › Images_examples_v20260307_1507/Others/Others (21).jpg]

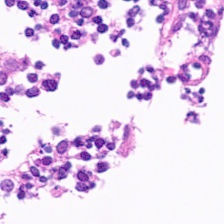

Supplement: Supplementary file 1 [file biomedicines-14-01134-s001.zip › Images_examples_v20260307_1507/Others/Others (22).jpg]

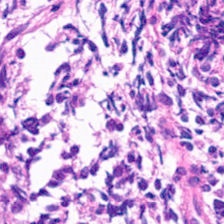

Supplement: Supplementary file 1 [file biomedicines-14-01134-s001.zip › Images_examples_v20260307_1507/Others/Others (23).jpg]

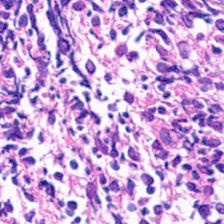

Supplement: Supplementary file 1 [file biomedicines-14-01134-s001.zip › Images_examples_v20260307_1507/Others/Others (24).jpg]

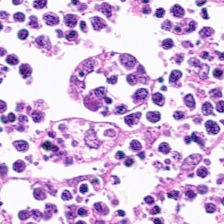

Supplement: Supplementary file 1 [file biomedicines-14-01134-s001.zip › Images_examples_v20260307_1507/Others/Others (25).jpg]

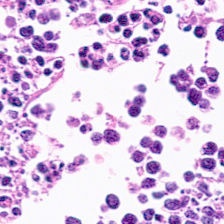

Supplement: Supplementary file 1 [file biomedicines-14-01134-s001.zip › Images_examples_v20260307_1507/Others/Others (26).jpg]

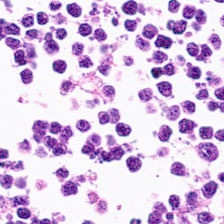

Supplement: Supplementary file 1 [file biomedicines-14-01134-s001.zip › Images_examples_v20260307_1507/Others/Others (27).jpg]

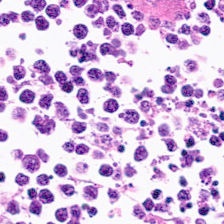

Supplement: Supplementary file 1 [file biomedicines-14-01134-s001.zip › Images_examples_v20260307_1507/Others/Others (28).jpg]

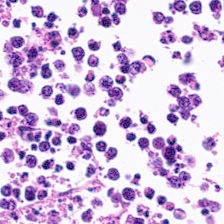

Supplement: Supplementary file 1 [file biomedicines-14-01134-s001.zip › Images_examples_v20260307_1507/Others/Others (29).jpg]

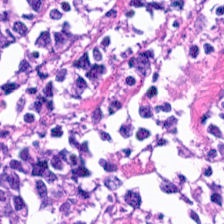

Supplement: Supplementary file 1 [file biomedicines-14-01134-s001.zip › Images_examples_v20260307_1507/Others/Others (3).jpg]

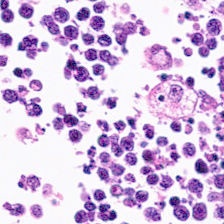

Supplement: Supplementary file 1 [file biomedicines-14-01134-s001.zip › Images_examples_v20260307_1507/Others/Others (30).jpg]

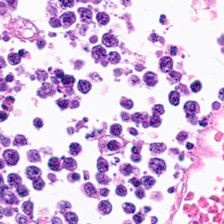

Supplement: Supplementary file 1 [file biomedicines-14-01134-s001.zip › Images_examples_v20260307_1507/Others/Others (31).jpg]

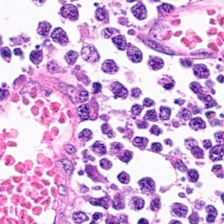

Supplement: Supplementary file 1 [file biomedicines-14-01134-s001.zip › Images_examples_v20260307_1507/Others/Others (32).jpg]

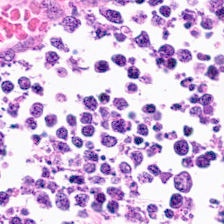

Supplement: Supplementary file 1 [file biomedicines-14-01134-s001.zip › Images_examples_v20260307_1507/Others/Others (33).jpg]

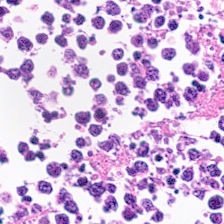

Supplement: Supplementary file 1 [file biomedicines-14-01134-s001.zip › Images_examples_v20260307_1507/Others/Others (34).jpg]

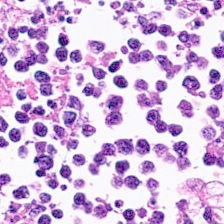

Supplement: Supplementary file 1 [file biomedicines-14-01134-s001.zip › Images_examples_v20260307_1507/Others/Others (35).jpg]

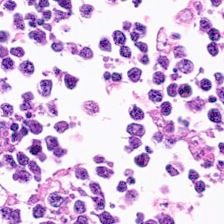

Supplement: Supplementary file 1 [file biomedicines-14-01134-s001.zip › Images_examples_v20260307_1507/Others/Others (36).jpg]

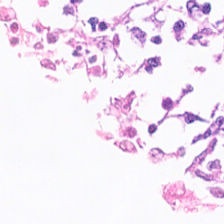

Supplement: Supplementary file 1 [file biomedicines-14-01134-s001.zip › Images_examples_v20260307_1507/Others/Others (37).jpg]

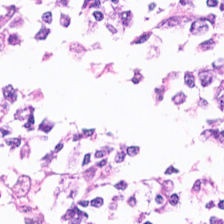

Supplement: Supplementary file 1 [file biomedicines-14-01134-s001.zip › Images_examples_v20260307_1507/Others/Others (38).jpg]

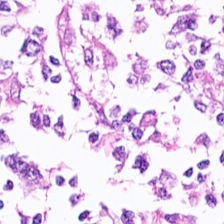

Supplement: Supplementary file 1 [file biomedicines-14-01134-s001.zip › Images_examples_v20260307_1507/Others/Others (39).jpg]

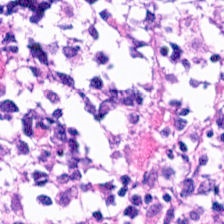

Supplement: Supplementary file 1 [file biomedicines-14-01134-s001.zip › Images_examples_v20260307_1507/Others/Others (4).jpg]

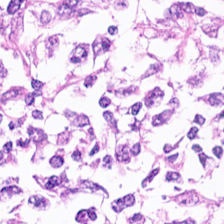

Supplement: Supplementary file 1 [file biomedicines-14-01134-s001.zip › Images_examples_v20260307_1507/Others/Others (40).jpg]

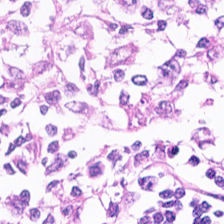

Supplement: Supplementary file 1 [file biomedicines-14-01134-s001.zip › Images_examples_v20260307_1507/Others/Others (41).jpg]

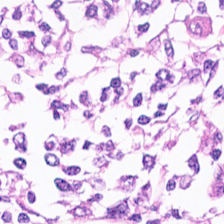

Supplement: Supplementary file 1 [file biomedicines-14-01134-s001.zip › Images_examples_v20260307_1507/Others/Others (42).jpg]

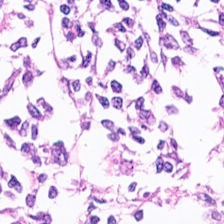

Supplement: Supplementary file 1 [file biomedicines-14-01134-s001.zip › Images_examples_v20260307_1507/Others/Others (43).jpg]

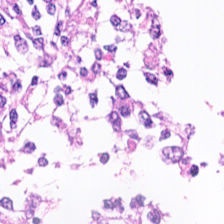

Supplement: Supplementary file 1 [file biomedicines-14-01134-s001.zip › Images_examples_v20260307_1507/Others/Others (44).jpg]

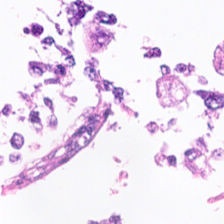

Supplement: Supplementary file 1 [file biomedicines-14-01134-s001.zip › Images_examples_v20260307_1507/Others/Others (45).jpg]
